# Therapeutic Applications of Immunobiologics in Autoimmune and Inflammatory Diseases

**DOI:** 10.3390/pharmaceutics18080917

**Published:** 2026-07-25

**Authors:** Kannan Badri Narayanan

**Affiliations:** 1 School of Chemical Engineering, Yeungnam University, 280 Daehak-Ro, Gyeongsan 38541, Gyeongsangbuk-do, Republic of Korea; okbadri@gmail.com or okbadri@yu.ac.kr; 2 Research Institute of Cell Culture, Yeungnam University, 280 Daehak-Ro, Gyeongsan 38541, Gyeongsangbuk-do, Republic of Korea

**Keywords:** autoimmune disease, inflammatory disease, biologics, cytokines, interleukin, monoclonal antibody, therapy

## Abstract

Immunobiologics or biologics have revolutionized the therapeutic paradigm of autoimmune and inflammatory diseases by selectively targeting and modulating dysregulated immune pathways. Compared with conventional broad-spectrum immunosuppressants, biologics provide a more specific and mechanism-based rational approach, often associated with improved efficacy and reduced adverse events. This review provides a comprehensive overview of the repertoire of therapeutic biologics, their mechanisms of action, pivotal clinical trials, and clinical applications. We discuss biologics targeting T-cell activation, depletion, and adhesion, as well as agents neutralizing pro-inflammatory cytokines, including tumor necrosis factor (TNF), interleukin (IL)-1, IL-6, IL-12, IL-17, IL-22, and IL-23, in addition to B-cell-directed therapies and IgE-modulating agents. Evidence from randomized clinical trials and observational studies highlights the profound therapeutic impact of these agents across a broad spectrum of immune-mediated diseases, including rheumatoid arthritis, psoriasis, systemic lupus erythematosus (SLE), ankylosing spondylitis, chronic spontaneous urticaria, asthma, ulcerative colitis, Crohn’s disease, and other forms of inflammatory bowel disease (IBD). Pharmacodynamic parameters, including receptor-binding affinity and downstream pathway modulation, are critical determinants of therapeutic efficacy, whereas pharmacovigilance remains indispensable for monitoring risks such as immunogenicity, opportunistic infections, and manufacturing-related variability. Continuous regulatory oversight by agencies such as the United States Food and Drug Administration (FDA) and the European Medicines Agency (EMA) safeguards standards of safety, efficacy, and quality. In parallel, the expanding development of biosimilars offers a key opportunity to enhance affordability and broaden global access to biologic therapies. Despite substantial clinical progress, important challenges persist, including disease heterogeneity, variability in therapeutic response, long-term safety concerns, and issues of cost-effectiveness. Future directions emphasize precision medicine strategies, including biomarker-guided treatment, rationally designed biologic combinations, next-generation antibody engineering, and the development of high-quality biosimilars to improve therapeutic durability, safety, and accessibility equity. By integrating mechanistic insights with clinical outcomes, this review underscores the transformative role of immunobiologics and delineates strategies to optimize their application in the management of autoimmune and inflammatory diseases.

## 1. Introduction

Autoimmune and inflammatory diseases are diverse and complex groups of chronic, relapsing, and often debilitating disorders that occur when the immune system becomes overactive or misdirected, attacking the body’s own tissues or responding inappropriately to persistent inflammatory stimuli [1,2]. Conditions such as rheumatoid arthritis, systemic lupus erythematosus, multiple sclerosis, inflammatory bowel disease, and psoriasis affect millions of people globally, contributing to morbidity, reduced quality of life, and growing healthcare expenditure [3,4]. Although substantial research efforts have been undertaken, the fundamental pathophysiological processes of these diseases are still only partly elucidated. Nonetheless, advances in fields like drug delivery, molecular biology, and immunology over the past three decades have transformed therapeutic strategies, enabling the development of interventions that selectively target specific components of the immune system rather than relying solely on broad-spectrum immunosuppressants [5,6].

In the past, the treatment of autoimmune and inflammatory diseases mainly depended on these non-specific agents, such as corticosteroids, non-steroidal anti-inflammatory drugs (NSAIDs), and conventional disease-modifying antirheumatic drugs (DMARDs) such as methotrexate, azathioprine, and cyclophosphamide [7,8]. While these therapies have been effective in controlling symptoms and delaying disease progression, they are often limited by incomplete disease control, adverse effects, and cumulative toxicity. Importantly, their broad immunosuppressive activity predisposes patients to opportunistic infections and malignancies, underscoring the need for more precise and safer therapeutic strategies [9]. With increasing insights into the immune pathways underlying these diseases, researchers have developed immunobiologics, engineered proteins such as monoclonal antibodies, soluble receptors, and fusion proteins, designed to selectively target specific immune cells, receptors, cytokines, or signaling molecules. Immunobiologics, commonly referred to as biologics, have revolutionized modern medicine by enabling a more targeted modulation of the immune system, interfering with discrete pathogenic pathways while preserving overall host defense [10]. For instance, tumor necrosis factor (TNF) inhibitors have transformed the treatment of rheumatoid arthritis, ankylosing spondylitis, and inflammatory bowel disease, converting previously severe conditions into manageable chronic disorders. Similarly, biologics targeting interleukin (IL)-17 or IL-23 pathways have demonstrated unprecedented efficacy in psoriasis, while B-cell depletion therapies, such as rituximab, have redefined management of systemic autoimmune conditions like lupus nephritis and vasculitis [11,12].

The advent of biologics has not only expanded therapeutic options but also provided critical insights into disease heterogeneity. Variability in patient responses to biologics, such as anti-TNF agents, revealed that individuals with the same clinical diagnosis may exhibit distinct underlying disease mechanisms [13]. This has prompted the investigation of novel therapeutic targets, including co-stimulatory pathways, adhesion molecules, and cytokine networks beyond TNF. Nonetheless, the widespread use of biologics has raised important considerations regarding long-term safety, immunogenicity, and high treatment costs. Challenges such as the development of neutralizing antibodies, increased susceptibility to infections, and rare but serious adverse events continue to be central concerns for both clinicians and patients [14]. From a scientific perspective, biologics represent a paradigm shift in translational research, bridging bench discoveries with effective bedside therapies for patients. The development of therapeutic monoclonal antibodies now constitutes a cornerstone of pharmaceutical pipelines [15]. Advances in genetic engineering, hybridoma technology, antibody humanization, and recombinant protein design have expedited the approval of several biologic agents for clinical use [16]. In parallel, the rapidly growing field of biosimilars, biologic products highly similar in structure and function to approved biologics, has emerged as a strategy to enhance patient access and reduce treatment costs, particularly in low- and middle-income countries [17].

Nevertheless, the field of immunobiologics is continuously evolving to respond effectively to the needs of all patients. Ongoing research into the development of next-generation biologics and combination regimens has improved pharmacokinetics and pharmacodynamics, particularly through the integration of biologics with various small-molecule agents and synthetic drugs [18]. These advances aim to personalize biologic therapy, guided by biomarkers; however, reliable prediction of treatment response and adverse effects remains elusive and requires long-term studies. Despite these challenges, personalized medicine approaches hold the promise for tailoring therapy to individual immunological profiles, thereby optimizing efficacy while minimizing risks [19].

A comprehensive literature search was conducted across PubMed, Scopus, and Web of Science, supplemented by ClinicalTrials.gov and regulatory and trial-outcome data from the FDA and EMA public assessment and label databases. The search ranged from January 2000 to November 2025, prioritizing publications from the past five years (2021–2025) for clinical trial updates, biosimilar approvals, and safety data, while foundational mechanistic and historical approval literature was drawn from the full period. Search strategies utilized combinations of each biologic’s international nonproprietary name (INN) and developmental code with specific disease terms (such as rheumatoid arthritis, psoriasis, inflammatory bowel disease, and systemic lupus erythematosus), mechanistic targets (such as TNF-α, IL-17, and IL-6 receptor), and study-type qualifiers (such as randomized controlled trial, phase III, biosimilar, immunogenicity, and pharmacovigilance). Inclusion was limited to peer-reviewed, English-language publications; conference abstracts without full-text data were excluded. Pivotal trials were selected based on their citation in FDA/EMA approval labels or their identification as primary registrational phase II/III studies within the drug’s regulatory history, which was further supplemented by selecting the highest-enrollment or most frequently cited trial when multiple studies were available.

With numerous biologics already approved and many others under clinical evaluation, a comprehensive review of their therapeutic applications across autoimmune and inflammatory diseases is imperative. Most available comprehensive reviews on immunobiologics are organized either by a single disease (such as rheumatoid arthritis or psoriasis alone) or by a single cytokine family. In contrast, this review utilizes a nomenclature architecture (mAb vs. fusion protein vs. recombinant protein vs. engineered fragment, of the WHO INN nomenclature hierarchy) and applies this analytical paradigm across seven distinct immune-pathway strategies and more than fifteen disease indications. This approach allows for the comparison of mechanistically analogous agents across multiple diseases rather than within a single diagnosis. This review focuses on the major immune-modulatory strategies employed by biologics, including targeting T-cell activation and adhesion, cytokine-directed therapies, and B-cell and IgE-targeted interventions. It also addresses underlying mechanisms of action, key clinical trial outcomes, and commercialization aspects, while elucidating future directions in the development of next-generation biologics.

## 2. Overview of Autoimmune and Inflammatory Diseases

Autoimmune and inflammatory diseases constitute a heterogeneous group of disorders characterized by aberrant immune responses directed against self-antigens or dysregulated inflammatory cascades that lead to chronic tissue damage. In autoimmune diseases, both central and peripheral immune tolerance mechanisms become compromised, leading to the inappropriate recognition of autologous antigens as non-self and subsequent activation of autoreactive T and B lymphocytes. Inflammatory diseases, while sharing overlapping pathophysiological features, may additionally arise from excessive or poorly regulated immune responses to exogenous antigens, pathogen-associated molecular patterns (PAMPs), or damage-associated molecular patterns (DAMPs) through dysregulated innate immune signaling pathways [20,21]. Collectively, these pathological conditions affect 5–10% of the global population, contributing substantially to morbidity, functional disability, and socioeconomic healthcare expenditure. For instance, rheumatoid arthritis (RA) affects approximately 1% of the population worldwide, while systemic lupus erythematosus (SLE) has a prevalence of 20–150 people per 100,000 individuals, depending on ethnicity and geography [22]. Inflammatory bowel diseases (IBD), including Crohn’s disease and ulcerative colitis, show a rising incidence in newly industrialized countries, reflecting the influence of environmental factors such as diet patterns, microbiome alterations, and urbanization [23].

The underlying immunopathogenesis involves complex interactions between both innate and adaptive immunity. Innate immune effector cells, including tissue-resident macrophages, circulating neutrophils, and antigen-presenting dendritic cells, function as primary initiators of inflammatory responses through the production and secretion of pro-inflammatory mediators such as interleukin-1β (IL-1β), tumor necrosis factor-α (TNF-α), and interferon-γ (INF-γ). Adaptive immune cells, particularly CD4+ and CD8+ T lymphocytes and antibody-producing B lymphocytes, subsequently sustain and amplify chronic immune activation through antigen-specific recognition and memory responses. The coordinated release of cytokines, chemokines, and upregulation of cellular adhesion molecules orchestrates the recruitment, extravasation, and activation of effector immune cells within target tissues, ultimately resulting in persistent inflammatory injury and progressive tissue remodeling [3,4,24,25].

Nonetheless, the pathogenetic mechanisms involved in autoimmune and inflammatory diseases are multifactorial, involving genetic predisposition, epigenetic regulation, environmental factors, and immune dysregulation. This requires sophisticated therapeutic approaches that address multiple pathways precisely and simultaneously. Conventional therapies, including corticosteroids, disease-modifying antirheumatic drugs (DMARDs), and immunosuppressants such as methotrexate, azathioprine, and cyclophosphamide, remain widely used as first-line treatments [26]. However, these therapeutic modalities have significant limitations as broad-spectrum immunosuppressive agents. Major concerns include increased susceptibility to opportunistic infections and malignancies, while long-term corticosteroid administration leads to osteoporosis, diabetes, and cardiovascular complications. Additionally, inadequate therapeutic response rates and disease relapses following drug withdrawal highlight the need for more targeted therapies with improved efficacy and safety profiles [27,28].

In response to these limitations, cytokines implicated in inflammatory processes, including interleukin-1 (IL-1), IL-6, IL-12, IL-17, IL-18, IL-23, tumor necrosis factor-α (TNF-α), and interferon-gamma (IFN-γ), are increasingly recognized as specific therapeutic targets in autoimmune and inflammatory diseases such as inflammatory bowel disease (IBD) and psoriatic arthritis [29,30]. Contemporary treatment strategies have evolved from conventional broad-spectrum immunomodulatory agents, including glucocorticoids, methotrexate, and statins, towards precision medicine approaches. The emergence of biologics, particularly anti-cytokine monoclonal antibodies, and small-molecule inhibitors such as Janus kinase (JAK) inhibitors and sphingosine-1-phosphate (S1P) modulators, enables more precise modulation of inflammatory cascades. These targeted therapeutic interventions represent a paradigm shift toward personalized, mechanism-based therapeutic interventions that offer improved therapeutic outcomes while minimizing systemic immunosuppressive effects [31,32,33].

## 3. Biologics for Autoimmune and Inflammatory Diseases

Biologic agents or biologics, broadly defined as pharmacologically active proteins derived from living systems or produced through recombinant technology, have fundamentally transformed the therapeutic landscape of autoimmune and inflammatory disorders. The advent of recombinant DNA technology has enabled large-scale synthesis of these complex molecules, ensuring both target specificity and enhanced safety profiles compared to conventional immunosuppressive agents. Contemporary advances in biotechnology have facilitated the development of “designer biologics” that selectively target discrete molecular pathways implicated in disease pathogenesis, exemplified by novel therapies for diseases that selectively inhibit pathogenic cytokines or receptor-ligand interactions [34,35].

Based on their structural characteristics and mechanisms of action, biologics employed in autoimmune and inflammatory diseases can be categorized into three main classes:

1. **Monoclonal antibodies (mAb):** These engineered immunoglobulin G (IgG) molecules demonstrate exquisite specificity for target antigens, including cell surface markers, soluble mediators, and cytokine receptors. The nomenclature reflects their degree of humanization: chimeric antibodies contain human constant regions (Fc) fused to murine variable regions (Fab), while humanized antibodies retain only minimal murine sequences within the complementarity-determining regions of the antigen-binding sites, thereby reducing immunogenicity [35].
2. **Fusion proteins (-cept):** These engineered molecules combine functional domains from distinct proteins to create novel therapeutic entities. One approach involves fusing human proteins to cytotoxic moieties, enabling antibody-mediated cellular internalization and subsequent intracellular cytotoxicity. Alternatively, soluble receptor constructs incorporate human receptor extracellular domains fused to immunoglobulin Fc fragments, effectively functioning as cytokine traps that sequester and neutralize soluble ligands, thereby preventing ligand-receptor interactions [36,37].
3. **Recombinant human proteins (-rh):** These agents represent either full-length human proteins or biologically active fragments produced in prokaryotic or eukaryotic expression systems, including bacterial, yeast, or mammalian cell cultures. These therapeutics typically function as receptor agonists, antagonists, or enzyme replacement therapies.

Currently, the majority of biologics require parenteral administration through subcutaneous, intramuscular, or intravenous routes due to their protein nature and susceptibility to gastrointestinal degradation. However, innovative drug delivery technologies, including oral formulations with protective carriers and topical preparations for localized disorders, are under active investigation to improve patient convenience and treatment adherence [38,39].

Therapeutic strategies employing biologics in autoimmune and inflammatory diseases are designed to interfere with key immunopathogenic mechanisms through multiple complementary approaches [10,40]:

1. T-cell activation inhibition: Targeting co-stimulatory pathways (CD28-B7, CD40-CD40L) or cytokine signaling cascades (IL-2 receptor blockade) to prevent T-cell priming and expansion.
2. T-cell depletion: Eliminating activated T lymphocytes through direct cytotoxicity, apoptosis induction, or antibody-dependent cell-mediated cytotoxicity (ADCC) mechanisms.
3. Leukocyte trafficking inhibition: Preventing T-lymphocyte extravasation and tissue infiltration by targeting adhesion molecules (selectins, ICAM-1) or integrins (α4β7, α4β1) essential for endothelial transmigration.
4. Cytokine inhibition/neutralization: Inhibiting or neutralizing pro-inflammatory mediators through specific antagonists, including anti-TNF-α, anti-IL-1, anti-IL-6, anti-IL-17, anti-IL-12/23 antibodies, and cytokine trap technologies.
5. Anti-inflammatory modulation: Administering recombinant anti-inflammatory cytokines, receptor antagonists, or agents that promote immunodeviation from Th1/Th17 toward Th2/Treg phenotypes.
6. B-cell targeted therapies: Implementing B-cell depletion strategies or inhibiting B-cell survival factors to reduce autoantibody production and antigen presentation capacity.
7. Allergic response modulation: Employing anti-IgE therapy to interrupt type I hypersensitivity reactions and mast cell degranulation in allergic inflammatory conditions.

These therapeutic strategies selectively modulate inflammatory cascades while maintaining critical immune functions.

## 4. Biologics Structure and Their International Nonproprietary Names (INNs)

**Figure 1:**
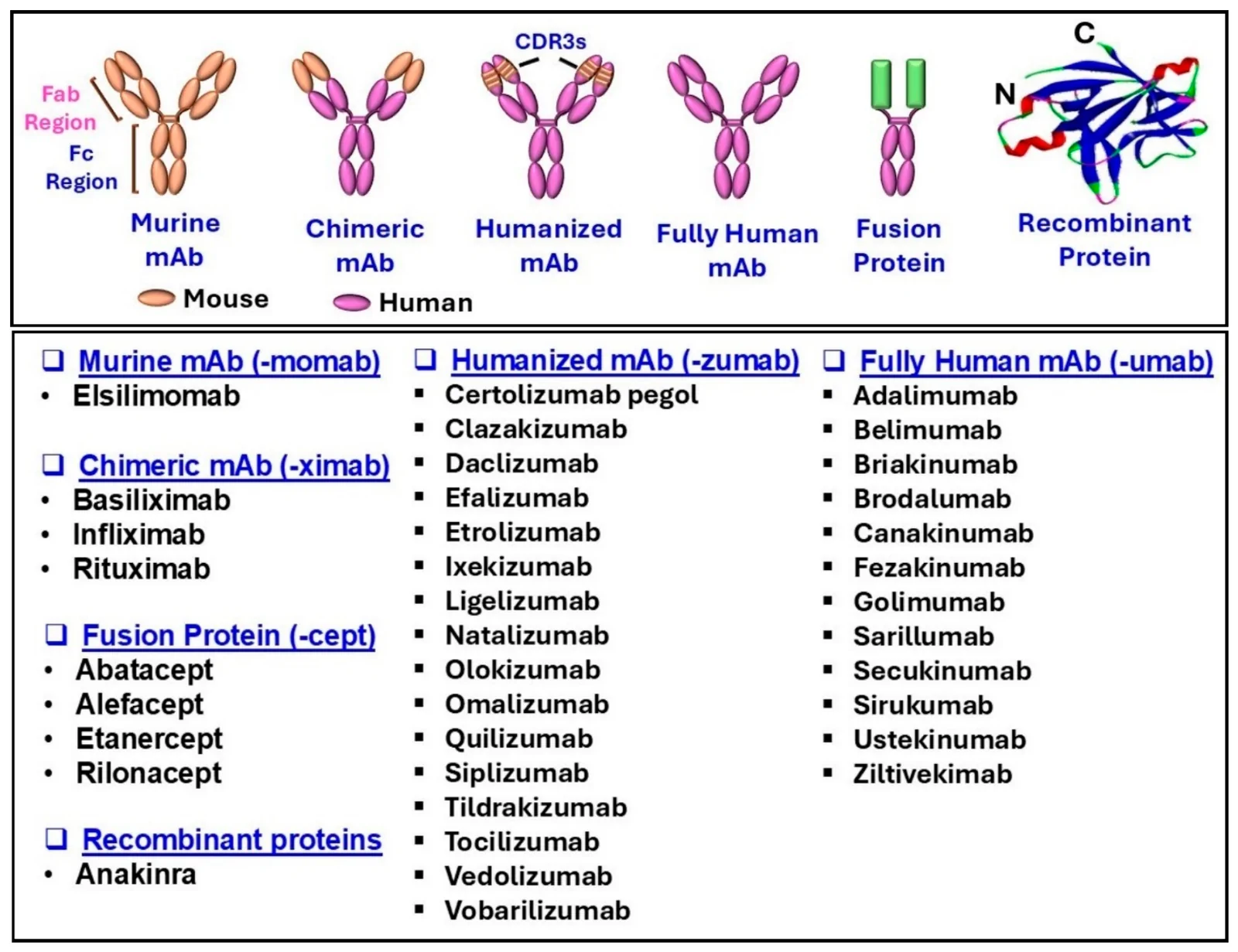
Classification of biologics and International Nonproprietary Name (INN) nomenclature.

In 1953, the World Health Organization (WHO) established the International Nonproprietary Names (INNs) system to provide a standardized, globally recognized nomenclature for pharmaceutical substances, including biologics. This systematic nomenclature incorporates specific stems and substems that convey critical information about the molecular structure, immunological origin, and therapeutic target of each biologic agent, ensuring consistency and clarity in both scientific and clinical contexts [41,42] (Figure 1).

### 4.1. Biologics Classification and Nomenclature

Biologic therapeutics used for autoimmune and inflammatory diseases are structurally categorized into four major classes: monoclonal antibodies, fusion proteins, recombinant proteins, and engineered antibody fragments.

#### 4.1.1. Monoclonal Antibodies

Monoclonal antibodies (mAbs) represent the largest class of biologics, typically consisting of immunoglobulin G (IgG) molecules with two heavy and two light chains that form a Y-shaped tetrameric structure. The fragment antigen-binding (Fab) regions contain variable domains that determine target specificity, whereas the fragment crystallizable (Fc) region mediates effector functions, including complement activation, antibody-dependent cellular cytotoxicity (ADCC), and prolonged serum half-life through neonatal Fc receptor (FcRn) binding.

The International Nonproprietary Name (INN) nomenclature incorporates a suffix system indicating antibody origin and humanization degree: (a) -omab (murine monoclonal antibodies): contains 100% murine sequences. These exhibit high immunogenicity, frequently eliciting human anti-mouse antibodies (HAMA), limiting therapeutic utility. (b) -ximab (chimeric monoclonal antibodies): comprise approximately 65–75% human and 25–35% murine sequences, with human constant domains and murine variable regions. Chimeric antibodies demonstrate reduced immunogenicity but may elicit human anti-chimeric antibodies (HACA). (c) –zumab (humanized monoclonal antibodies): incorporate 90–95% human sequences with only complementarity-determining regions (CDRs) or minimal murine framework sequences retained. These are generated through CDR grafting techniques, which significantly reduce immunogenicity. (d) –umab (fully human monoclonal antibodies): contain entirely human-derived sequences generated through transgenic mouse technology, phage display libraries, or human hybridoma technology with minimized immunogenicity [43,44].

The INN nomenclature follows the pattern [prefix] + [target infix] + [source infix] + [stem (-mab)]. The target infix indicates the pharmacological target class, for example, -li- (immune system), -ki- (interleukin-related), -ne- (nervous system), and -tu- (tumor). The source inflix indicates the antibody’s origin, for example, -o- (murine), -xi- (chimeric), -zu- (humanized), -u- (fully human). The stem “-mab” denotes monoclonal antibody [45].

#### 4.1.2. Fusion Proteins

Fusion proteins are bioengineered molecules that combine functional domains from distinct proteins and are designated with the stem -cept. These typically consist of (a) receptor-Ig fusion constructs: extracellular receptor domain fused to IgG1 Fc regions, forming soluble decoys that sequester target ligands. For instance, etanercept fuses TNFR2 extracellular domains with IgG1 Fc, functioning as a TNF-α/β trap; (b) co-stimulatory molecule-Ig constructs: abatacept fuses the CTLA-4 extracellular domain with modified IgG1 Fc, binding CD80/CD86 on antigen-presenting cells to block CD28-mediated T-cell co-stimulation; and (c) immunoadhesion constructs: alefacept fused LFA-3 with IgG1 Fc, binding CD2 on T lymphocytes to inhibit their activation and induce selective apoptosis [46,47,48].

#### 4.1.3. Recombinant Proteins

Recombinant proteins are naturally occurring human proteins produced using recombinant DNA technology and function as cytokine receptor antagonists or immunomodulatory mediators. For example, anakinra is a recombinant non-glycosylated human IL-1 receptor antagonist (IL-1Ra) consisting of 153 amino acids with an N-terminal methionine from *Escherichia coli* expression [49,50].

#### 4.1.4. Engineered Antibody Fragments

Engineered antibody fragments are modified portions of antibodies that retain the antigen-binding region (Fab) but lack part or all of the Fc region. These fragments can be Fab (fragment antigen-binding), scFv (single-chain variable fragment), or F(ab’)_2_, nanobodies, or other engineered constructs. Certolizumab pegol (CZP) is a PEGylated (conjugated with polyethylene glycol) Fab’ fragment of a humanized monoclonal antibody. The lack of an Fc domain eliminates complement-dependent cytotoxicity [51,52].

## 5. Biologics in the Management of Autoimmune and Inflammatory Diseases

### 5.1. Biologics Targeting T-Cell Activation (Co-Stimulatory Blockade or IL-2 Signaling Inhibition)

**Figure 2:**
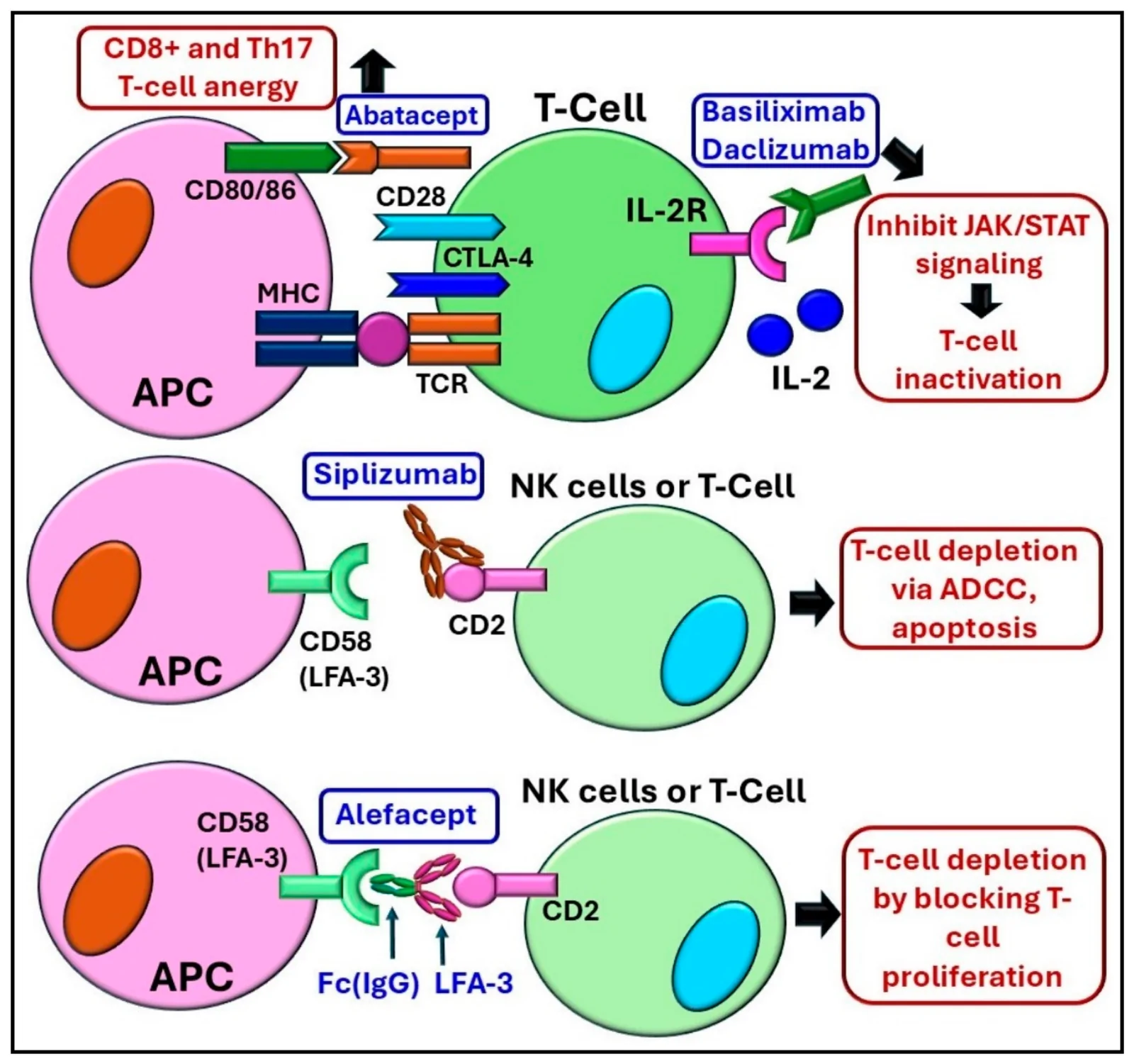
Mechanisms of action of biologics targeting T-cell activation and inducing depletion of activated T-cells.

T-cell activation requires not only antigen recognition through the T-cell receptor but also a second co-stimulatory signal mediated by CD28 binding to CD80/CD86 on antigen-presenting cells. This co-stimulatory pathway is central to immune tolerance, immune response initiation, and the pathogenesis of autoimmune inflammation. Persistent or aberrant T-cell activation sustains chronic inflammatory diseases such as RA, psoriatic arthritis (PsA), and skin psoriasis, making blockade of the CD28-CD80/86 axis an attractive therapeutic strategy [53,54] (Figure 2) (Table 1 and Table 2).

#### 5.1.1. Abatacept (ABA)

Abatacept is a fully recombinant human fusion protein composed of the extracellular domain of cytotoxic T-lymphocyte antigen 4 (CTLA-4/CD152) linked to the Fc portion of human IgG1. Modulation of the CD28/CD80/CTLA-4 pathway can downregulate T-cell activation and subsequent inflammatory responses. This mechanism is therapeutically exploited in the treatment of autoimmune diseases characterized by T-cell-mediated inflammation [55]. By competitively binding CD80/CD86, abatacept prevents CD28-mediated T-cell co-stimulation, thereby attenuating downstream T-cell activation, cytokine release, and tissue-damaging inflammation [56].

The U.S. Food and Drug Administration granted initial approval for abatacept on 23 December 2005, under the trade name Orencia (Bristol-Myers Squibb, New York, NY, USA), for adults with moderate-to-severe rheumatoid arthritis (RA), Juvenile idiopathic arthritis (JIA), and psoriatic arthritis (PsA) patients refractory to disease-modifying antirheumatic drugs (DMARDs), such as methotrexate or TNF inhibitors. Evidence also suggests its utility in rarer inflammatory conditions such as Juvenile Dermatomyositis (JDM), which is a rare inflammatory myopathy affecting skin and proximal muscles. Abateacept combined with sodium thiosulfate has been successfully used in refractory JDM, leading to the reduction in ulcerated muscle and skin inflammation and attenuation of calcinosis progression [57]. Beyond autoimmune diseases, co-stimulation blockade may influence cardiovascular inflammation. Anti-TNFα and anti-IL-6R monoclonal antibodies have shown cardioprotective effects, and anti-IL-1β therapy significantly reduced major cardiovascular events in coronary artery disease [58]. Similarly, preclinical studies demonstrated that abatacept reduced myocardial injury by inhibiting MAPK activation and enhancing Nrf2/HO-1 signaling, thereby improving ventricular function in rat models of myocardial infarction [59]. In atherosclerosis, abatacept-mediated CD28-CD80/86 blockade has been shown to reduce inflammatory activation and cardiovascular risk [60].

Abatacept has also been evaluated in allergic and myopathic conditions. In a murine model of allergic rhinitis, abatacept reduced IL-4, IL-5, IL-13, IL-17A, and IFN-γ levels in nasal tissues, suppressing Th2-driven inflammation [61]. Clinical and preclinical data further support its benefit in idiopathic inflammatory myopathies (IIMs) such as polymyositis, dermatomyositis, and inclusion body myositis [62]. In murine models of cardiac diseases, abatacept enhanced the production of anti-inflammatory cytokine IL-10 and reduced T cell and macrophage infiltration into cardiac tissue, thereby limiting myocardial damage and improving functional outcomes [63].

Interestingly, abatacept also modulates regulatory T-cell (Treg) activity. Nakachi et al. (2017) demonstrated that treatment enhanced the proportion of LAG3^+^ Tregs, which are enriched in IL-10-producing subsets, thereby contributing to immunoregulation [64]. Furthermore, abatacept suppresses autoantibody-driven cytokine production. In monocytes exposed to anti-citrullinated protein antibodies (ACPA) and rheumatoid factor (RF), abatacept inhibited the upregulation of TNF-α, IL-1β, IL-6, IL-8, and CCL2 by inducing indoleamine 2,3-dioxygenase (IDO), enhancing its anti-inflammatory effects in seropositive autoimmune disease [65]. The drug’s immunomodulatory activity extends to skeletal and joint health. While CTLA-4Ig favors bone formation under physiological conditions, excessive immunosuppression may impair bone regeneration in pathological states [66]. Combination regimens have been tested to optimize efficacy: abatacept with dexamethasone, and particularly with dexamethasone plus methotrexate, showed superior suppression of macrophage cytokine production compared with monotherapies [67].

Although generally well-tolerated, rare adverse effects have been reported. Almogairen (2019) described abatacept-induced granulomatous allergic hepatitis with sarcoid-like granulomatous pathology in murine models, highlighting the importance of intensive monitoring [68]. Collectively, abatacept illustrates the clinical relevance of co-stimulation blockade in autoimmune and inflammatory disorders. By targeting a central immunological checkpoint, it not only improves outcomes in RA, PsA, and JIA but also shows promise in cardiovascular disease, allergic disorders, and inflammatory myopathies.

#### 5.1.2. Basiliximab (BAS)

Activation and clonal expansion of T cells are critically dependent on IL-2 signaling. The IL-2 receptor consists of three subunits: the α-chain (CD25, p55), β-chain (CD122, p75), and the common γ-chain (CD132). CD25 is transiently upregulated following T-cell activation, making it a selective target for immunomodulation. Basiliximab is a chimeric murine-human IgG1κ monoclonal antibody directed against the IL-2 receptor α-chain (CD25; IL-2Rα). By binding CD25, it prevents IL-2 from interacting with its receptor, thereby inhibiting downstream signaling required for the proliferation of activated T cells [69,70]. Clinically, basiliximab is primarily employed to prevent acute rejection in organ transplantation, particularly kidney transplants [71]. By selectively blocking the IL-2 pathway, it reduces pathogenic T-cell activation while preserving resting lymphocytes, thereby minimizing broad immunosuppression. Basiliximab is marketed under the trade name Simulect^®^. It was developed by Novartis Pharmaceuticals and remains an important component of induction therapy in transplantation medicine [72].

#### 5.1.3. Daclizumab (DAC)

Daclizumab is a humanized IgG1 monoclonal antibody that targets the IL-2 receptor α-chain (CD25), thereby preventing IL-2-mediated T-cell activation and increasing IL-2 bioavailability, promoting expansion of CD56^bright^ natural killer (NK) cells [73]. Initially approved by the U.S. FDA for preventing acute rejection in renal transplantation, Daclizumab (previously marketed as Zenapax^®^) was later explored for its immunomodulatory potential in autoimmune conditions, including psoriasis. In a phase II clinical trial for psoriasis, daclizumab was administered at an initial dose of 2 mg/kg followed by 1 mg/kg biweekly infusions. However, its antipsoriatic efficacy was limited, showing benefit only in a subset of patients with active pustular psoriasis [74]. Beyond T-cell suppression, daclizumab exhibited secondary immunomodulatory effects, including the expansion of natural killer (NK) cells, which may contribute to its therapeutic profile. Despite its potential, daclizumab was withdrawn from the market in 2018 due to significant safety concerns. Reports of severe adverse effects, including life-threatening inflammatory brain disorders (e.g., encephalitis and meningoencephalitis), hepatotoxicity, and immune-mediated reactions such as lymphadenopathy, prompted its discontinuation [75].

### 5.2. Biologics Targeting Activated T-Cell Depletion

Biologics that deplete activated T cells employ mechanisms such as direct cytotoxicity, apoptosis induction, or antibody-dependent cellular toxicity (ADCC) and complement-dependent cytotoxicity (CDC). These agents target specific surface markers on T cells to reduce their numbers, thereby attenuating pathogenic immune responses. This approach is particularly relevant in autoimmune conditions like psoriasis, where T-cell-mediated inflammation drives disease pathology [76,77]. The biologics discussed below, alefacept and siplizumab, exemplify strategies to modulate or eliminate activated T cells, with distinct mechanisms and clinical outcomes. Alefacept and siplizumab both target the CD2/LFA-3 axis to modulate T-cell activity, with alefacept primarily inducing depletion via ADCC and siplizumab combining depletion with co-stimulatory blockade [78,79]. Both were developed for psoriasis, addressing the central role of T cells in its pathogenesis (Figure 2) (Table 1 and Table 2).

#### 5.2.1. Alefacept (ALE)

Alefacept is a fusion protein comprising the extracellular domain of lymphocyte function-associated antigen-3 (LFA-3) linked to the Fc portion of human IgG1. It primarily targets CD2-expressing memory effector T cells, which are upregulated in autoimmune inflammatory conditions such as psoriasis. By competitively binding to CD2, alefacept inhibits the LFA-3/CD2 interaction, thereby suppressing T-cell activation and proliferation. Additionally, the Fc portion of alefacept interacts with FcγRIII on NK cells, triggering antibody-dependent cellular cytotoxicity (ADCC) and selective depletion of pathogenic T cells [80,81].

Alefacept was the first biologic approved for moderate-to-severe chronic plaque psoriasis in adults, receiving FDA approval in February 2003 under the trade name Amevive^®^ by Astellas Pharma (formerly developed by Biogen Idec, Cambridge, MA, USA) [82]. Psoriasis is a T-cell-mediated autoimmune disorder characterized by keratinocyte hyperproliferation, epidermal hyperplasia, angiogenesis, and infiltration of CD4^+^ and CD8^+^ T cells producing inflammatory mediators such as IL-2, IL-8, and TNF-α. Alefacept therapy reduces activation of dermal CD4^+^ and epidermal CD8^+^ T cells, normalizes keratinocyte proliferation, and mitigates cutaneous inflammation [83,84]. Compared with other biologics used for psoriasis, alefacept exhibits a slower onset of action. Time to clinical improvement is approximately 15.4 weeks for high-dose alefacept, compared with 3.5–6.6 weeks for infliximab, adalimumab, etanercept, and ustekinumab [85]. Declining clinical use and market demand led to its discontinuation in 2011.

#### 5.2.2. Siplizumab (SIP)

Siplizumab is a humanized IgG1 monoclonal antibody directed against CD2, a surface glycoprotein expressed on T cells and natural killer (NK) cells. By targeting CD2, siplizumab disrupts the CD2-LFA-3 co-stimulatory pathway, thereby reducing T-cell activation and selectively depleting pathogenic T-cell populations. In clinical studies, subcutaneous or intravenous administration of Siplizumab at doses ranging from 0.04 mg/kg to 7 mg resulted in approximately a 50% reduction in peripheral memory and naïve T-cell counts in patients with moderate-to-severe psoriasis. An open-label, dose-escalation study over eight weeks demonstrated a 75% reduction in mean Psoriasis Area and Severity Index (PASI) in 50% of treated patients, with mild adverse effects including lymphopenia, chills, and headache [86,87]. Despite promising immunomodulatory activity, clinical development of siplizumab was discontinued in 2003 due to safety concerns related to immunosuppression and insufficient efficacy for regulatory approval.

### 5.3. Biologics Targeting T-Cell Adhesion and Extravasation (Integrin/Adhesion Molecule Blockade)

**Figure 3:**
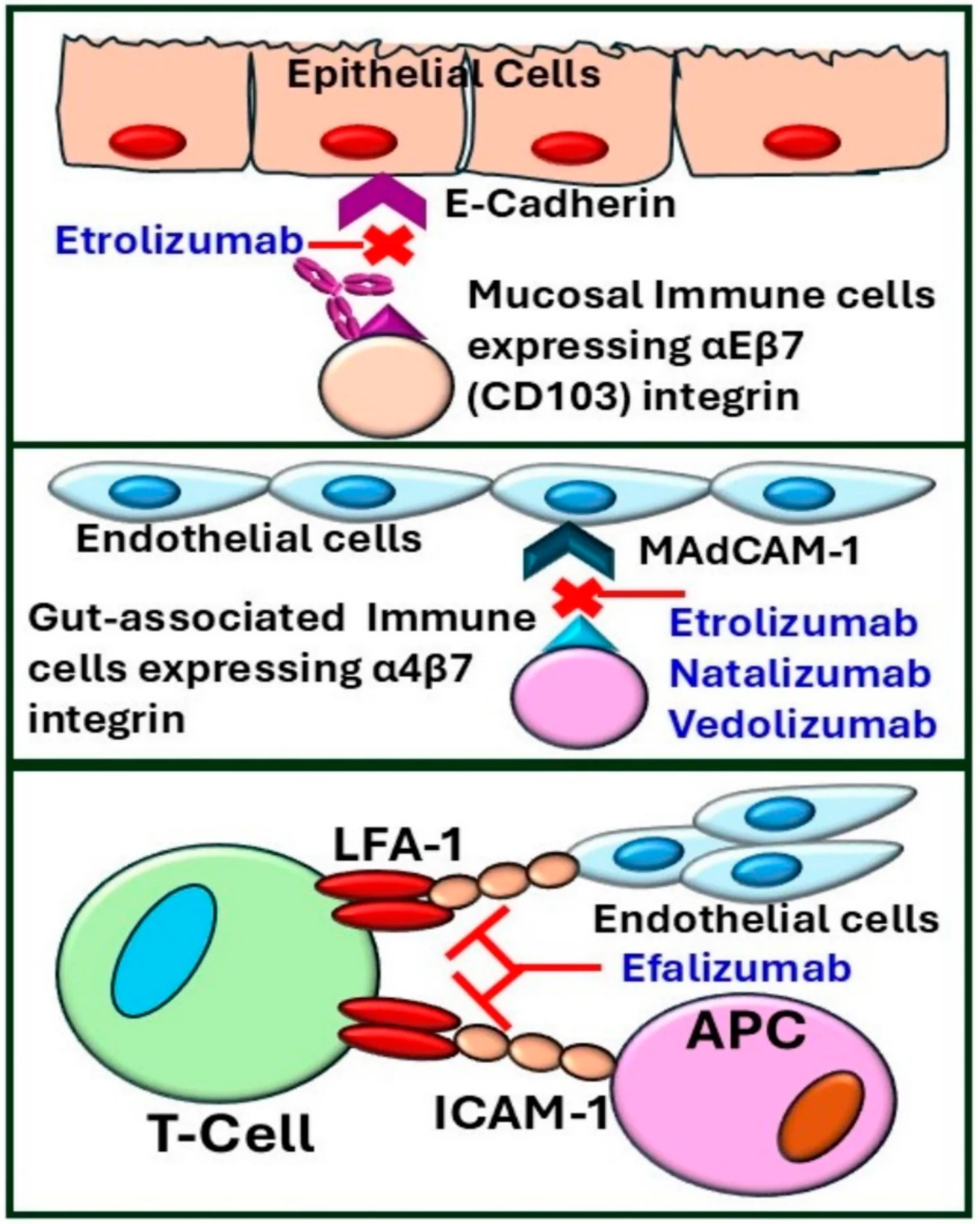
Mechanisms of action of biologics targeting T-cell adhesion and transendothelial migration (extravasation).

Biologics that inhibit T-cell adhesion and extravasation target integrins and adhesion molecules to prevent T-cell migration into inflamed tissues. By disrupting key interactions between T cells and endothelial or antigen-presenting cells (APCs), these agents mitigate immune-driven inflammation, particularly in autoimmune conditions like psoriasis [88,89] (Figure 3) (Table 1 and Table 2).

#### 5.3.1. Efalizumab (EFA)

Efalizumab is a humanized IgG1 monoclonal antibody directed against the alpha-1 subunit (CD11a) of leukocyte function-associated antigen-1 (LFA-1) on T cells. LFA-1 interacts with intracellular adhesion molecule-1 (ICAM-1) expressed on antigen-presenting cells (APCs), vascular endothelial cells, and keratinocytes. This interaction provides both a co-stimulatory signal for T-cell activation and a critical adhesion mechanism for T-cell migration into inflamed tissues. By binding to CD11a, efalizumab disrupts the LFA-1/ICAM-1 interaction, thereby inhibiting T-cell activation, adhesion, and extravasation into target tissues such as psoriatic skin [90].

Efalizumab received FDA approval in 2003 for subcutaneous treatment of moderate-to-severe chronic plaque psoriasis. While it effectively reduced T-cell-mediated cutaneous inflammation, early administration was occasionally associated with transient flu-like symptoms, including fever, myalgia, and malaise. However, long-term safety concerns emerged, particularly the increased risk of progressive multifocal leukoencephalopathy (PML), a rare but serious demyelinating brain infection caused by JC virus reactivation. Due to these significant safety concerns, efalizumab was voluntarily withdrawn from the U.S. market in 2009 [91]. Thus, efalizumab’s mechanism of therapeutic potential targeting T-cell adhesion molecules to control autoimmune inflammation by blocking LFA-1/ICAM-1 interactions effectively reduced T-cell-driven pathology in psoriasis. The withdrawal of efalizumab prompted a shift toward alternative biologics with improved safety profiles for psoriasis, such as those targeting IL-17 or IL-23 pathways [92,93].

#### 5.3.2. Etrolizumab (ETR)

Etrolizumab (rhuMAb Beta 7) is a recombinant humanized IgG1κ monoclonal antibody that binds to the β7 subunit of the α4β7 and αEβ7 integrin heterodimers. It represents a next-generation antiadhesion molecule developed as a gut-targeted therapy for inflammatory bowel disease (IBD), principally ulcerative colitis (UC) and Crohn’s disease (CD) [94,95]. Etrolizumab is the first investigational dual anti-integrin biologic, designed to target two critical pathways of intestinal inflammation by selectively inhibiting α4β7 and αEβ7. This dual inhibition regulates the trafficking of immune cells into the gut as well as their retention and pro-inflammatory activity within the intestinal mucosa. α4β7 is a key mediator of leucocyte homing to the gastrointestinal tract via interactions with mucosal addressin cell adhesion molecule-1 (MAdCAM-1) expressed on high endothelial venules in the gut mucosa [96,97,98]. α4β7 is expressed on monocytes, lymphocytes, eosinophils, basophils, progenitor mast cells, macrophages, and follicular dendritic cells, but is absent on neutrophils. In contrast, the αEβ7 integrin selectively binds to E-cadherin, mediating adhesion of intraepithelial lymphocytes (IELs) to intestinal epithelial cells [99]. Notably, >90% of IELs and approximately 50% of T cells in the lamina propria express αEβ7 integrin, underscoring its distinctive role in mucosal immunity.

By targeting the common β7 subunit, etrolizumab simultaneously blocks two complementary pathways: (i) inhibition of α4β7:MAdCAM-1 interactions reduces the recruitment of gut-homing T lymphocytes from the circulation into intestinal tissues, and (ii) inhibition of αEβ7:E-cadherin interactions diminishes the retention of lymphocytes within the epithelial compartment. Through this dual mechanism, impairing both trafficking and retention, etrolizumab is characterized as a gut-selective, dual-action anti-β7 therapy [98]. In a randomized, double-blind, placebo-controlled Phase I trial, etrolizumab demonstrated acceptable safety, tolerability, and pharmacodynamic activity in patients with moderate-to-severe UC [100]. The subsequent Phase II EUCALYPTUS study reported clinical benefit over placebo in moderate-to-severe UC [101].

The Phase III clinical development program was designed to evaluate the efficacy and safety of etrolizumab in patients with moderate-to-severe active UC or Crohn’s disease refractory or intolerant to corticosteroids, immunosuppressants, and/or anti-TNFs. This program comprised six multicenter, randomized controlled trials (RCTs) and two open-label extension (OLE) studies. The HIBISCUS I and II trials, multicenter, randomized, double-blind, placebo- and active-controlled studies, assessed induction of remission in moderate-to-severe UC. Etrolizumab demonstrated superiority to placebo in HIBISCUS I and was generally well tolerated [102]. The pivotal Phase III BERGAMOT trial in Crohn’s disease was initiated but later discontinued. In early 2022, Roche/Genentech announced termination of the CD program after mixed efficacy results, although certain induction endpoints were met, maintenance outcomes were inconsistent, and etrolizumab did not demonstrate superiority over established anti-TNF therapies [102,103]. Overall, while etrolizumab showed promise in select populations, the lack of consistent long-term efficacy led to discontinuation of its late-stage development program.

#### 5.3.3. Natalizumab (NAT)

Natalizumab is a humanized IgG4κ monoclonal antibody that selectively targets the α4 subunit of integrins on leukocytes, thereby inhibiting interactions of both α4β1 and α4β7 integrins. Through this mechanism, natalizumab prevents leukocyte adhesion and trans-endothelial migration of activated lymphocytes into target tissues such as the central nervous system (CNS) and gastrointestinal tract, thereby reducing tissue inflammation and lesion development [104]. It is the first α4 integrin antagonist and was approved by the U.S. FDA under the trade name TYSABRI^®^ (Biogen, Inc., Cambridge, MA, USA) for relapsing forms of multiple sclerosis (MS) and, subsequently, for certain cases of Crohn’s disease (CD). Although highly efficacious in relapsing MS, natalizumab carries a risk of progressive multifocal leukoencephalopathy (PML), a rare opportunistic demyelinating encephalopathy caused by reactivation of the John Cunningham (JC) virus. This necessitates rigorous risk stratification and mandatory enrollment in the TOUCH safety program [105].

The pivotal AFFIRM Phase III monotherapy trial, a randomized, double-blind, placebo-controlled study in relapsing MS, demonstrated a ~68% reduction in the annualized relapse rate at one year, with significant decreases in new MRI lesion accrual and risk of disability progression over two years [104]. The SENTINEL Phase III trial, in which natalizumab was administered in combination with interferon β-1a, showed additional reductions in relapse rate and some benefit on disability progression; however, the occurrence of two early PML cases in the combination arm contributed to major safety concerns, resulting in temporary market withdrawal in 2005. The drug was subsequently reintroduced in 2006 under the restricted TOUCH program [106]. Natalizumab received FDA approval in 2004 and EMA approval in 2006 for relapsing MS.

In Crohn’s disease, natalizumab was evaluated in the ENACT-1 (induction), ENACT-2 (maintenance), and ENCORE (confirmatory subgroup) Phase II/III trials. These trials demonstrated statistically significant induction and maintenance of clinical response and remission in selected patient subgroups, with modest overall absolute remission rates. Clinical benefit was particularly notable in patients with concomitant immunosuppressant therapy. Importantly, natalizumab induced and maintained responses in patients who were refractory to anti-TNF therapy. Nevertheless, systemic α4 blockade and the associated PML risk limited its widespread adoption in CD, and gut-selective anti-integrins were subsequently favored [107]. Natalizumab received FDA approval for moderate-to-severe CD in 2008, but EMA declined approval due to safety concerns. In August 2023, the U.S. FDA approved Tyruko^®^ (natalizumab-sztn), developed by Sandoz Inc. (Basel, Switzerland), as the first biosimilar to Tysabri for the treatment of adults with relapsing forms of multiple sclerosis [108].

#### 5.3.4. Vedolizumab (VDZ)

Vedolizumab (MLN0002) is a humanized IgG1 monoclonal antibody that selectively targets the α4β7 integrin, thereby inhibiting its interaction with MAdCAM-1 (mucosal addressin cell adhesion molecule-1) expressed on gut endothelial venules. This blockade prevents the adhesion and extravasation of α4β7-expressing gut-homing T lymphocytes into intestinal tissue, thereby reducing intestinal inflammation [109]. Unlike natalizumab, vedolizumab exhibits high specificity for α4β7 and does not bind to, nor inhibit the function of, α4β1 or αEβ7 integrins. Consequently, it selectively interferes with T-cell adhesion to MAdCAM-1 without affecting vascular cell adhesion molecule-1 (VCAM-1) interactions. Vedolizumab has been approved for the treatment of moderate-to-severe ulcerative colitis (UC) and Crohn’s disease (CD) and is marketed as Entyvio^®^ (Takeda Pharmaceuticals, Cambridge, MA, USA) for both intravenous infusion and subcutaneous administration [110]. Because vedolizumab selectively blocks α4β7 and not α4β1, it does not broadly impair CNS immune surveillance. Consistently, no causal association with PML, a risk predominantly linked to α4β1 blockade, has been established in randomized clinical trials or post-marketing surveillance of vedolizumab [109].

The pivotal Phase II/III GEMINI clinical development program provided the primary evidence for regulatory approval. In UC, the Phase III GEMINI 1 randomized, placebo-controlled induction and maintenance trials demonstrated that vedolizumab was significantly superior to placebo in inducing clinical response and remission, as well as in maintaining remission through Week 52 in moderate-to-severe UC patients [110]. In CD, the Phase III GEMINI 2 trials demonstrated that vedolizumab achieved higher rates of clinical remission at Week 52 compared with placebo. However, the induction benefit at Week 6 was more modest overall, with stronger signals observed in specific subgroups, such as TNF-naïve patients [111]. Thus, GEMINI 2 confirmed vedolizumab’s efficacy for maintenance therapy in CD, though its induction effect appeared less pronounced and often delayed relative to UC.

In patients with refractory CD and prior anti-TNF therapy failure, the GEMINI 3 trial—a Phase III, randomized, double-blind, placebo-controlled induction study—did not achieve its primary endpoint of clinical remission at Week 6 in the overall refractory population. Nevertheless, clinical benefit became apparent at later time points, suggesting that vedolizumab’s therapeutic effect in CD may emerge more gradually, particularly in TNF-experienced patients [112]. These findings support current clinical guidance that continuation of therapy beyond the early induction phase may be warranted before declaring treatment failure in certain CD populations.

### 5.4. Biologics Targeting Proinflammatory Cytokines and Mediators

A pivotal mechanism in immune-mediated inflammatory disorders involves the overproduction of cytokines such as TNF-α, IL-1, IL-6, IL-17, IL-12/23, and IL-22. Several biologics have been developed to neutralize these mediators and attenuate pathological immune responses. Among them, TNF-α inhibitors represent the earliest and most extensively studied class [113,114] (Table 1 and Table 2).

#### 5.4.1. Anti-TNF Agents as Biologics

**Figure 4:**
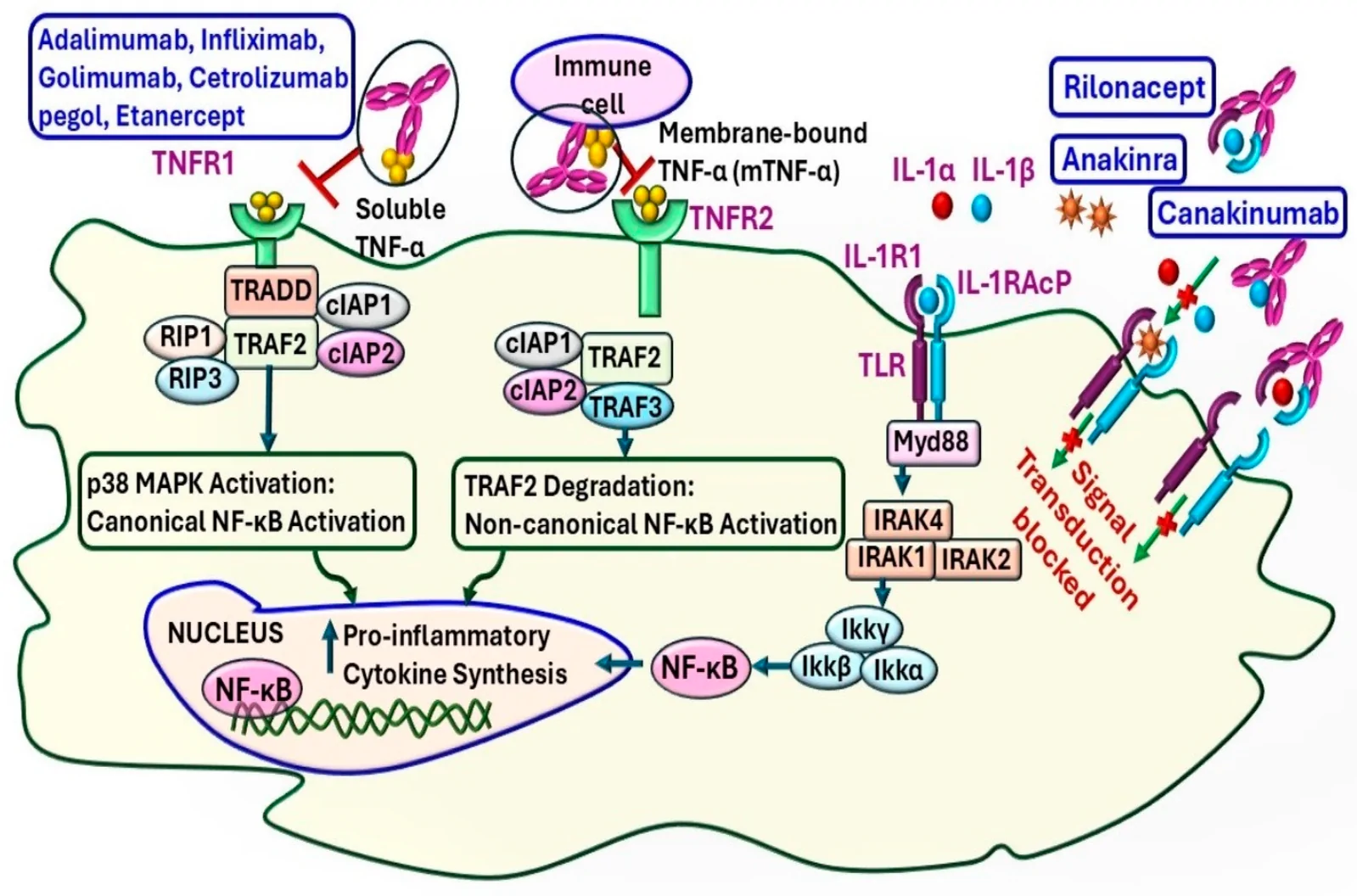
Mechanism of action of biologics targeting tumor necrosis factor-α (TNF-α), Interleukin-1 (IL-1), and IL-1 receptors within proinflammatory cytokine networks.

TNF-α promotes nuclear factor kappa B (NF-κB) activation, leading to the production of pro-inflammatory cytokines (IL-1, IL-6, and IL-8) and upregulation of adhesion molecules on endothelial cells, which contributes to leukocyte migration and tissue inflammation [115,116]. Therapies targeting tumor necrosis factor-alpha (TNF-α) represent a proven approach for managing autoimmune and inflammatory conditions, notably rheumatoid arthritis (RA), psoriasis, and inflammatory bowel disease (IBD). Clinically available options include full IgG1 monoclonal antibodies such as infliximab, adalimumab, and golimumab; the PEGylated Fab’ fragment certolizumab pegol; and the TNF receptor 2 (TNFR2) extracellular domain fused to IgG1-Fc, known as etanercept [117] (Figure 4).

##### Adalimumab (ADA)

Adalimumab, a fully humanized IgG1 monoclonal antibody, targets TNF-α, a key cytokine in systemic inflammation [118]. By binding TNF-α, it prevents interaction with TNF receptors, thereby reducing inflammatory responses in autoimmune diseases. Approved by the U.S. Food and Drug Administration in December 2002 as Humira^®^, adalimumab has a half-life of 10–20 days and is indicated for moderate-to-severe RA, psoriatic arthritis (PsA), plaque psoriasis, Crohn’s disease, ulcerative colitis, ankylosing spondylitis, and juvenile idiopathic arthritis (JIA). It downregulates proinflammatory cytokines, including IL-6, IL-8, and GM-CSF, alleviating symptoms such as joint swelling in RA, gut inflammation in IBD, and skin lesions in psoriasis [119,120,121]. In psoriasis, affecting 1–3% of the global population, with plaque psoriasis comprising 70–80% of cases, adalimumab significantly reduces disease severity [122]. Long-term treatment (1 year) improved Psoriasis Area and Severity Index (PASI), Dermatology Life Quality Index (DLQI), and Nail Psoriasis Severity Index (NAPSI) scores, enhancing quality of life. In IBD, adalimumab induces and maintains remission in Crohn’s disease and ulcerative colitis [123]. A notable case involved successful treatment of Crohn’s disease triggered post-chemotherapy for pelvic paraganglioma [124].

Adalimumab’s versatility extends to rare conditions. In polyarteritis nodosa (PAN), a vasculitis causing necrotizing inflammation of medium and small arteries, adalimumab mitigated symptoms in hepatitis B-negative PAN patients [125]. For birdshot chorioretinopathy, a rare ocular autoimmune disorder, biweekly 40 mg subcutaneous injections improved visual acuity [126]. In sporadic Blau syndrome, caused by NOD2 gene mutations and characterized by granulomatous arthritis, uveitis, and skin lesions, adalimumab resolved treatment-resistant cutaneous manifestations [127]. Similarly, in neurosarcoidosis, adalimumab reduced granulomatous inflammation affecting the nervous system [128]. In Vogt-Koyanagi-Harada (VKH) syndrome, a multisystem autoimmune disorder involving the eyes, skin, ears, and central nervous system, adalimumab effectively controlled inflammation without corticosteroids [129]. For adult-onset Still’s disease (AOSD), a systemic inflammatory disorder with fever, arthritis, and rashes, adalimumab served as an effective alternative therapy [130]. In Rasmussen’s encephalitis (RE), a rare chronic inflammatory brain disorder causing epilepsy and neurologic deficits, adalimumab reduced seizure frequency without neurocognitive decline.

Adalimumab also shows promise in hidradenitis suppurativa (HS), a chronic inflammatory skin condition with painful nodules and sinus tracts. Treatment for 12 weeks increased anti-inflammatory macrophages (CD206^+^), though plasma IL-6 and hsCRP levels increased, with improvements in DLQI scores [131]. In actinic granuloma and multicentric reticulohistiocytosis (MRH), a rare non-Langerhans cell histiocytosis, adalimumab resolved granulomatous inflammation, often in combination with methotrexate and prednisolone [132]. In Takayasu arteritis (TAK), adalimumab controlled refractory scleritis, a rare ocular manifestation of this large-vessel granulomatous vasculitis affecting the aorta, its major branches, and the pulmonary arteries [133]. Safety concerns include rare instances of acute autoimmune leukoencephalopathy. In major depressive disorder (MDD) with elevated inflammatory biomarkers, adalimumab, adjunctive to sertraline, improved Hamilton Depression Rating Scale (HAM-D) scores and reduced inflammation [134]. In proliferative vitreoretinopathy (PVR), adalimumab did not induce inflammatory or fibrogenic gene expression in retinal pigment epithelial cells, suggesting safety in ocular applications [135]. While adalimumab is generally well tolerated, rare adverse events include autoimmune demyelinating disorders, such as acute leukoencephalopathy. The availability of adalimumab biosimilars, such as ZRC-3197 developed by Cadila Healthcare (Zydus Cadila, India), which are structurally and functionally highly similar to adalimumab and demonstrate comparable clinical outcomes, expands global access to this therapy [136].

##### Certolizumab Pegol (CZP)

Certolizumab pegol is a PEGylated Fab’ fragment of a humanized anti-TNF-α monoclonal antibody [137]. Unlike adalimumab and infliximab, it lacks the IgG1 Fc region, thereby avoiding Fc-mediated functions such as antibody-dependent cellular cytotoxicity (ADCC) and complement-dependent cytotoxicity (CDC). The conjugation of polyethylene glycol (PEG) extends its plasma half-life and improves pharmacokinetics. Certolizumab pegol has demonstrated efficacy in Crohn’s disease, rheumatoid arthritis, and psoriatic arthritis [138,139,140]. In certolizumab pegol, the absence of an Fc region minimizes the potential for Fc-mediated immune activation while preserving potent anti-inflammatory activity.

##### Etanercept (ETN)

Etanercept is a recombinant soluble TNF-α receptor fusion protein composed of two extracellular domains of the TNF receptor fused to the Fc portion of a human IgG antibody. It has been approved by the US FDA and EMA for the treatment of RA, psoriatic arthritis, plaque psoriasis, polyarticular juvenile idiopathic arthritis, and ankylosing spondylitis [141]. In 2002 and 2004, the FDA extended its approval to include psoriatic arthritis and psoriasis, respectively. Etanercept acts by binding circulating TNF-α, thereby preventing its interactions with cell surface TNF receptors. This reduces the circulating TNF and mitigates the TNF-mediated inflammation in diseases such as RA and psoriasis. The recommended dose is 25 mg administered subcutaneously twice weekly [142].

In a double-blind, dose-escalation study of psoriasis patients, Leonardi et al. (2003) [143] reported that 44% of patients treated with 50 mg etanercept twice a week showed at least a 75% improvement in the PASI. Since moderate-to-severe psoriasis is associated with systemic inflammation, it constitutes an independent risk factor for cardiovascular disease [143]. Anti-inflammatory treatments, including biologics, have been suggested to reduce cardiovascular risk factors and inflammatory markers. However, systemic therapies with adalimumab, infliximab, etanercept, efalizumab, or alefacept in combination with ultraviolet (UV) B phototherapy did not significantly affect overall cardiovascular risk [144].

In addition, a phase II clinical trial of etanercept (50 mg/week subcutaneously) in patients with moderate-to-severe hidradenitis suppurativa (HS) demonstrated minimal evidence of clinically significant efficacy at this dosage [145]. The adverse effects of etanercept are comparable to those of other TNF-α inhibitors and include increased susceptibility to infections and injection-site reactions [146]. Autoantibody formation has also been reported, including antinuclear and anti-dsDNA antibodies, with clinical manifestations such as drug-induced lupus, discoid lupus erythematosus, and necrotizing vasculitis related to cryoglobulinemia. Furthermore, cases of candidal septicemia and bilateral chlorioretinitis have been described in HS patients receiving etanercept and corticosteroids in the presence of indwelling intravenous catheters [147,148].

##### Golimumab (GOL)

Golimumab is a fully human IgG1κ monoclonal antibody that selectively targets TNF-α. It binds with high affinity and specificity to both soluble and transmembrane forms of TNF-α, neutralizing their bioactivity by preventing interaction with TNF-α receptors. This blockade inhibits downstream inflammatory signaling pathways, thereby reducing immune-mediated tissue damage [140]. Golimumab is commercially available under the brand name Simponi^®^ and is manufactured by Janssen Biotech Inc. (Horsham, PA, USA). It has received regulatory approval for the treatment of several autoimmune diseases, including rheumatoid arthritis, psoriatic arthritis, ankylosing spondylitis, and ulcerative colitis [149].

##### Infliximab (IFX)

Infliximab is a chimeric mouse-human IgG1 monoclonal antibody that specifically targets TNF-α, a key pro-inflammatory cytokine involved in the pathogenesis of multiple autoimmune and inflammatory disorders. Infliximab is approved for the treatment of rheumatoid arthritis (RA), Crohn’s disease (CD), ulcerative colitis (UC), ankylosing spondylitis (AS), and psoriatic arthritis. It is also effective in managing extraintestinal manifestations of IBD, including IBD-associated arthropathy, a subset of spondyloarthropathy (SpA) [150]. Tenascin-C (TNC), an extracellular matrix glycoprotein expressed in response to inflammation and tissue damage, participates in IBD and Crohn’s disease and is associated with response to infliximab therapy. Elevated serum and mucosal Tenascin-C (TNC) levels in UC and CD correlate with poor response to infliximab therapy, suggesting TNC as a potential biomarker [151]. In pediatric CD, infliximab therapy increases circulating adiponectin, an anti-inflammatory adipokine that regulates insulin sensitivity and mediates protective metabolic effects [152].

Infliximab infusions have improved clinical outcomes in Crohn’s disease and demonstrated efficacy in acute necrotizing pancreatitis by reducing serum markers of oxidative stress and inflammation, including sialic acid, malondialdehyde, carbonyl content, and amylase, while enhancing total antioxidant activity [153]. It has also been employed in refractory sarcoidosis [154] and contributes to ameliorating inflammatory eye disorders such as Behcet’s disease-related uveitis and peripheral ulcerative keratitis (PUK), improving both visual outcomes and reducing ocular inflammation [155,156]. In an ex vivo porcine model of retinal degeneration, infliximab attenuated Zaprinast-induced retinal degeneration, which arises from inhibition of cGMP-degrading phosphodiesterase 6 (PDE6), indicating infliximab’s neuroprotective potential [157].

In moderate-to-severe UC, combination therapy with infliximab and azathioprine induced higher rates of corticosteroid-free remission and mucosal healing at 16 weeks compared to monotherapy [158]. Kawasaki disease (KD) is an acute inflammation of blood vessels (vasculitis), particularly the coronary arteries. In Kawasaki disease (KD) patients unresponsive to intravenous immunoglobulin (IVIG) therapy, infliximab effectively reduced coronary artery lesions [159]. In another instance, corticosteroid-resistant ipilimumab-induced colitis in patients with unresectable or metastatic disease was managed with infliximab, which exerts its therapeutic effect by suppressing T-cell activation and inhibiting pro-inflammatory cytokines such as IL-1 and IL-6 [160]. However, rare adverse events, including interstitial pneumonitis, have been reported [161].

Infliximab exerts an anti-inflammatory effect not only by direct TNF-α neutralization but also via induction of host cell apoptosis [162]. Spray-dried formulations of infliximab offer potential for local administration via the respiratory tract to suppress lung inflammation by inhibiting lung-secreted TNF-α [163]. Clinical and genetic markers, such as ZNF133, a zinc finger protein, have been associated with differential patient response to infliximab in IBD, reflecting its immunomodulatory effects [164]. In RA, combination therapy with methotrexate and infliximab improves high-density lipoprotein (HDL) functionality, underscoring the contribution of TNF-α to cardiovascular risk modulation [165]. In an ex vivo study, Gertel et al. (2024) [166] investigated the effects of IFX and MTX on peripheral blood mononuclear cells (PBMCs) and synovial fluid mononuclear cells (SFMCs) from patients with inflammatory arthritis. Both agents effectively inhibited PBMC proliferation and IFN-γ production. However, only infliximab reduced synovial monocytes and suppressed pro-inflammatory gene expression in SFMCs [166]. The first infliximab biosimilar, CT-P13 (Remsima^®^, Inflectra^®^), exhibits comparable structural, physicochemical, and biological characteristics to the originator, providing a cost-effective alternative for RA and IBD treatment [167,168]. SB2 (Renflexis^®^, Flixabi^®^ in Europe), a second biosimilar developed by Samsung Bioepis Co., Ltd. (Incheon, Republic of Korea), was approved by the EMA in 2016 and the FDA in 2017 for RA treatment [169].

#### 5.4.2. Anti-IL-1/IL-1 Pathway Targeted Biologics

Interleukin-1 (IL-1), comprising IL-1α and IL-1β, is a key pro-inflammatory cytokine that orchestrates innate and adaptive immune responses [170]. Dysregulated IL-1 signaling contributes to the initiation and amplification of inflammation by promoting leukocyte recruitment, upregulating adhesion molecules, and inducing downstream cytokines and chemokines. Excessive IL-1 activity has been implicated in the pathogenesis of several autoimmune and autoinflammatory diseases, including rheumatoid arthritis, systemic juvenile idiopathic arthritis, adult-onset Still’s disease, and cryopyrin-associated periodic syndromes [171,172].

Biologic agents targeting the IL-1/IL-1 receptor axis have been developed to counteract this pathogenic signaling. These include recombinant receptor antagonists, monoclonal antibodies against IL-1 isoforms, and soluble decoy receptors, which collectively act to neutralize IL-1 activity or block receptor-mediated signaling. By specifically inhibiting IL-1-driven pathways, these biologics provide effective therapeutic strategies for controlling chronic inflammation and limiting immune-mediated tissue damage in diverse inflammatory and autoimmune disorders. Three pharmacologic inhibitors of IL-1 are commercially available: anakinra, canakinumab, and rilonacept [173] (Figure 4) (Table 1 and Table 2).

##### Anakinra (ANR)

Anakinra (Kineret^®^, Swedish Orphan Biovitrum, Stockholm, Sweden) is a recombinant, nonglycosylated form of human interleukin-1 receptor antagonist (rhIL-1ra). It mimics the endogenous IL-1Ra, competitively blocking IL-1α and IL-1β from binding to IL-1 receptors, thereby preventing recruitment of the IL-1 receptor accessory protein (IL-1RAcP) and subsequent inflammatory signaling. Through this mechanism, it functions as an anti-inflammatory cytokine analog of the naturally occurring IL-1Ra. Anakinra was the first anti-IL-1 biologic approved by the U.S. FDA (2001), followed by approval in the European Union and Canada, and is indicated for disorders such as rheumatoid arthritis (RA) and cryopyrin-associated periodic syndromes [174,175,176,177].

IL-1 is central to the pathogenesis of RA, promoting synovial inflammation, pannus formation, cartilage destruction, and bone resorption in various animal models. Anakinra has demonstrated clinical efficacy in patients with moderate-to-severe RA unresponsive to conventional disease-modifying antirheumatic drug (DMARD) therapy, significantly reducing oxidative stress and serum IL-6 levels [178,179,180]. In the European Union, anakinra is approved for use in combination with the DMARD methotrexate to alleviate the signs and symptoms of RA in patients with inadequate response to methotrexate monotherapy [181]. IL-1 also plays a pivotal role in systemic-onset juvenile idiopathic arthritis (sJIA). Subcutaneous administration of anakinra (2–10 mg/kg/day) has demonstrated significant clinical benefits in affected children [182]. In a randomized clinical trial, RA patients treated with high-dose hr-ILRa (150 mg/day) showed reduced macrophages in the intimal and subintimal layers, along with decreased lymphocyte infiltration. Treatment also led to a 74% reduction in E-selectin expression on synovial vascular endothelium, and diminished VCAM-1 and ICAM-1 expression on infiltrating mononuclear cells in both layers [183].

IL-1 contributes significantly to cardiovascular disease (CVD) by promoting IL-6 production, vascular inflammation, and prothrombotic activity. On a systemic level, IL-1 stimulates the production of IL-6 and enhances pro-inflammatory and pro-coagulant events, resulting in a prothrombotic environment that further exacerbates adverse cardiovascular events. Histological studies by Dewberry et al. (2000) revealed the presence of IL-1β in human atherosclerotic plaques, indicating its significance in CVD [184]. Moreover, IL-1 is crucial in the initiation and progression of abdominal aorta aneurysms. The blockade of the IL-1 receptor type 1 (IL-1R1) using anakinra effectively attenuates aneurysm formation in mouse models [185]. Furthermore, anakinra has been observed to downregulate C-reactive protein (CRP) and IL-6 in patients following myocardial infarction. It has also demonstrated efficacy in improving microvascular dysfunction associated with ischemic stroke, hemorrhagic stroke, diabetes, and coronary artery disease (CAD). In a randomized clinical trial, the treatment with anakinra resulted in a 29% enhancement of coronary flow reserve (CFR) and an 18.7% improvement in global longitudinal strain after three months of therapy [186].

Beyond rheumatology and cardiology, anakinra has demonstrated therapeutic benefit across a broad spectrum of inflammatory conditions. In non-rheumatic secondary hemophagocytic lymphohistiocytosis (sHLH), subcutaneous administration of anakinra (6–10 mg/kg/day) combined with dexamethasone achieved clinical remission without significant adverse effects [187]. Dermatological and autoinflammatory manifestations, including isotretinoin-induced acne fulminans and synovitis, acne, pustulosis, hyperostosis, and osteitis (SAPHO) syndrome, have shown rapid improvement with combination therapy involving anakinra, bisphosphonates, and corticosteroids [188]. In Majeed syndrome, a rare autosomal recessive disorder associated with *LPIN2* gene mutations, which encodes the phosphatidic acid phosphatase enzyme, and characterized by chronic multifocal osteomyelitis and congenital dyserythropoietic anemia, anakinra (1.5 mg/kg/day) is considered an effective anti-inflammatory intervention [189,190].

Neuroinflammation has been implicated in the onset and recurrence of seizures by increasing neuronal excitability during status epilepticus (SE). SE is characterized by the failure of mechanisms that terminate seizures or the initiation of pathways that lead to abnormally prolonged seizures. This condition can potentially result in long-term consequences, including neuronal death, neuronal injury, and impaired neuronal network functions [191]. The use of anti-cytokine therapy shows promise in treating SE and drug-resistant epilepsies. Specifically, IL-1β signal blockade with Anakinra has demonstrated efficacy in reducing seizure recurrence and generalization in patients with inflammatory refractory epilepsy [192]. Moreover, anakinra serves as a potent anti-inflammatory agent to reduce mortality in COVID-19 patients exhibiting elevated inflammatory biomarkers, as it can lower levels of C-reactive protein (CRP), serum ferritin, and serum d-dimer levels [193].

##### Canakinumab (CAN)

Canakinumab (ACZ885, Ilaris; Novartis, Basel, Switzerland) is a fully humanized IgGκ monoclonal antibody (mAb) that selectively neutralizes IL-1β, while also targeting the N-terminal truncated isoform of IL-1α (NotIL-1α) [194]. By inhibiting IL-1β signaling, canakinumab modulates innate immune responses and exerts potent anti-inflammatory effects. In 2009, the US FDA and the European Medicines Agency (EMA) approved canakinumab as an orphan medicine for the treatment of multiple autoinflammatory conditions, including rheumatoid arthritis, cryopyrin-associated periodic syndromes (CAPS), Muckle-Wells syndrome (MWS), familial cold auto-inflammatory syndrome (FCAS), tumor necrosis factor receptor-associated periodic syndrome (TRAPS), hyperimmunoglobulin D syndrome (HIDS)/mevalonate kinase deficiency (MKD), familial Mediterranean fever (FMF), and systemic juvenile idiopathic arthritis (sJIA) [195]. CAPS represents a spectrum of rare inherited autoinflammatory diseases resulting from *NLRP3* mutations that affect cryopyrin, a protein central to acute inflammatory responses [196].

Gout is an inflammatory arthritis caused by monosodium urate (MSU) crystal deposition due to hyperuricemia, with a prevalence of ~4% in the United States. MSU crystals activate the NLRP3 inflammasome, leading to caspase-1 activation and subsequent IL-1β maturation, which promotes recruitment of synovial cells and phagocytes and drives acute joint inflammation [197,198]. Conventional treatments include NSAIDs, colchicine, and corticosteroids. However, Schlesinger (2012) demonstrated that canakinumab is superior to triamcinolone acetonide in acute gout and more effective than colchicine in gout flare prophylaxis, reducing both pain and the risk of new flares [199].

Canakinumab has an extended half-life, allowing subcutaneous administration at 150–300 mg every 4–8 weeks in adults (or 2–4 mg/kg in children). A phase II pilot study preceding the landmark Canakinumab Anti-inflammatory Thrombosis Outcomes Study (CANTOS) trial showed that a single 1.5 mg/kg dose reduced serum CRP by >40% for up to 12 weeks [28,200]. The CANTOS trial randomized post-myocardial infarction patients to receive canakinumab 50, 150, or 300 mg every three months, demonstrating dose-dependent reductions in CRP levels, with a primary endpoint of nonfatal MI, nonfatal stroke, or cardiovascular death [201]. Similarly, the randomized, placebo-controlled CANTOS trial demonstrated that canakinumab showed effectiveness in the treatment of atherosclerotic vascular lesions in patients to prevent secondary cardiovascular complications [202]. Additional analyses confirmed that blockade of IL-1β with canakinumab in patients with stable atherosclerotic CVD significantly lowered the risk of recurrent cardiovascular events [203]. Lorenzatti and Luz Servato (2018) further highlighted that these benefits were achieved without affecting LDL cholesterol levels, underscoring the anti-inflammatory rather than lipid-modifying mechanism of action [204].

Inflammation plays a central role in both coronary and peripheral atherosclerotic diseases, with elevated pro-inflammatory cytokines closely associated with cardiovascular risk. Canakinumab, a targeted anti-IL-1β therapy, has been investigated for atherosclerotic disease, including peripheral arterial disease. In randomized, double-blind trials, canakinumab significantly reduced systemic inflammation, as evidenced by decreases in plasma IL-6 and high-sensitivity C-reactive protein (hsCRP), without lowering LDL cholesterol levels, indicating a reduction in recurrent cardiovascular events in post-myocardial infarction patients [58,201]. Additionally, canakinumab has shown potential in the treatment of viral pericarditis caused by cardiotropic viruses, as well as in non-specific pericarditis mediated by inflammasome activation [205].

Another rare autoinflammatory disorder of autosomal recessive inheritance, known as hyperimmunoglobulin D syndrome (HIDS), caused by specific mutations in the mevalonate kinase (*MVK*) gene, is traditionally managed with NSAIDs, steroids, colchicine, TNF-α inhibitors, and anti-IL-1. Long-term therapy with canakinumab (300 mg every 4 weeks for 5 years) demonstrated sustained remission of fever and arthritis attacks in patients unresponsive to anakinra, with favorable tolerability [206]. Furthermore, canakinumab, administered at 2 mg/kg every 4 or 8 weeks, reduced inflammation associated with Majeed syndrome [190]. Subclinical inflammation in insulin-resistant tissues and impaired insulin secretion from pancreatic β-cells promote the initiation and progression of type 2 diabetes [207]. Hyperglycemia activates NOD-like receptor pyrin-3 (NLRP3) inflammasomes, leading to IL-1β activation and local autoinduction of IL-1 within pancreatic islets [208]. Clinical studies show that IL-1 blockade modestly reduces glycosylated hemoglobin (HbA1c) [209]. In a randomized trial of diabetic patients with prior MI and elevated hsCRP, subcutaneous administration of canakinumab (50–300 mg every 3 months) prevented major cardiovascular events and reduced fasting plasma and HbA1c and slowed the progression of type 2 diabetes during the first 6–9 months, though long-term metabolic benefits were inconsistent [210].

In schizophrenia, chronic inflammation has been implicated in disease progression. A recent randomized, double-blind, placebo-controlled trial of adjunctive subcutaneous canakinumab in chronically ill patients demonstrated reductions in serum hsCRP, a marker of peripheral inflammation, along with improvements in symptom severity [211]. Beyond neuroinflammation, CANTOS exploratory analyses revealed that patients receiving high-dose canakinumab (300 mg) had significant relative risk reductions in non-small-cell lung cancer (NSCLC) incidence (67%) and mortality (77%) [212,213,214]. Recently, Garon et al. (2024) conducted the CANOPY-A trial, a double-blind, randomized, placebo-controlled study evaluating canakinumab as adjuvant therapy in operable patients with stage II–IIIA or IIIB non-small-cell lung cancer (NSCLC) who had undergone complete resection and adjuvant cisplatin-based chemotherapy [215]. Treatment with canakinumab (200 mg every 3 weeks for 18 cycles) resulted in reductions in CRP and IL-6 levels but did not improve disease-free survival (DFS) or demonstrate correlation with clinical outcomes.

##### Rilonacept (RLC)

Rilonacept (also known as the “IL-1 trap”) is a novel dimeric fusion protein engineered to function as a soluble decoy receptor. It is composed of the extracellular domains of the interleukin-1 receptor type I (IL-1R1) and the IL-1 receptor accessory protein (IL-1RacP), fused to the Fc portion of human IgG1. By binding with high affinity to both IL-1α and IL-1β, rilonacept effectively neutralizes their biological activity and suppresses downstream IL-1-mediated inflammatory signaling [216].

Rilonacept was initially developed for the management of cryopyrin-associated periodic syndromes (CAPS), and its efficacy led to approval by the U.S. FDA under the “Orphan Drug Status” [217]. Clinical investigation has demonstrated that NSAIDs, corticosteroids, and antihistamines can alleviate CAPS-associated symptoms, whereas IL-1 inhibitors such as anakinra have significantly improved systemic inflammation and clinical outcomes. These findings supported subsequent investigations into rilonacept and canakinumab for long-term disease control and prevention of chronic inflammatory complications [218]. In early clinical development, a phase I trial demonstrated that rilonacept was well tolerated during six months of treatment in patients with recent-onset type 1 diabetes; however, it did not preserve β-cell function [219]. Beyond CAPS, rilonacept has also been investigated in several other inflammatory conditions. For instance, in subacromial bursitis—an inflammatory disorder of the bursa situated between the supraspinatus tendon and the overlying coracoacromial ligament and acromion—rilonacept, when administered with corticosteroid injections, demonstrated efficacy in reducing local inflammation [220]. Additionally, rilonacept was also evaluated in the SURGE trial, a double-blind, active-controlled study in which patients with acute gout were randomized within 48 h of symptom onset to receive indomethacin alone, rilonacept plus indomethacin, or rilonacept alone. The findings demonstrated that both rilonacept in combination with indomethacin and rilonacept monotherapy significantly improved ongoing gout-related pain and inflammation [221].

A major therapeutic advancement of rilonacept has been its application in the management of recurrent pericarditis (RP), a debilitating condition affecting approximately 15–30% of individuals following an initial episode of acute pericarditis. RP is associated with persistent or relapsing inflammation of the pericardial sac and contributes substantially to morbidity. Anticytokine biologics have emerged as promising anti-inflammatory therapies for RP, demonstrating favorable outcomes in a phase II trial evaluating their efficacy in patients with recurrent or refractory disease [28]. In a case report of a patient with severe ulcerative colitis complicated by RP, prolonged anti-inflammatory therapy with rilonacept, an IL-1 inhibitor, resulted in a marked reduction in pericardial inflammation [222]. The use of IL-1 blockade with anakinra, together with its favorable safety profile, supported the clinical development of rilonacept for patients with RP who are colchicine-resistant and glucocorticoid-dependent. This ultimately led to its approval by the U.S. FDA for the treatment of RP [223,224].

The efficacy of rilonacept was further evaluated in the RHAPSODY trial (Rilonacept Inhibition of Interleukin-1 Alpha and Beta for Recurrent Pericarditis: A Pivotal Symptomatology and Outcomes Study) by Gupta and colleagues (2021), which demonstrated that rilonacept is an effective therapeutic option capable of reducing the frequency of RP episodes and facilitating steroid tapering [225]. In a subsequent phase III trial, rilonacept significantly reduced the risk of pericarditis recurrence compared with placebo and promoted the rapid resolution of recurrent episodes [226].

#### 5.4.3. Anti-IL-17/IL-17 Receptor Biologics

**Figure 5:**
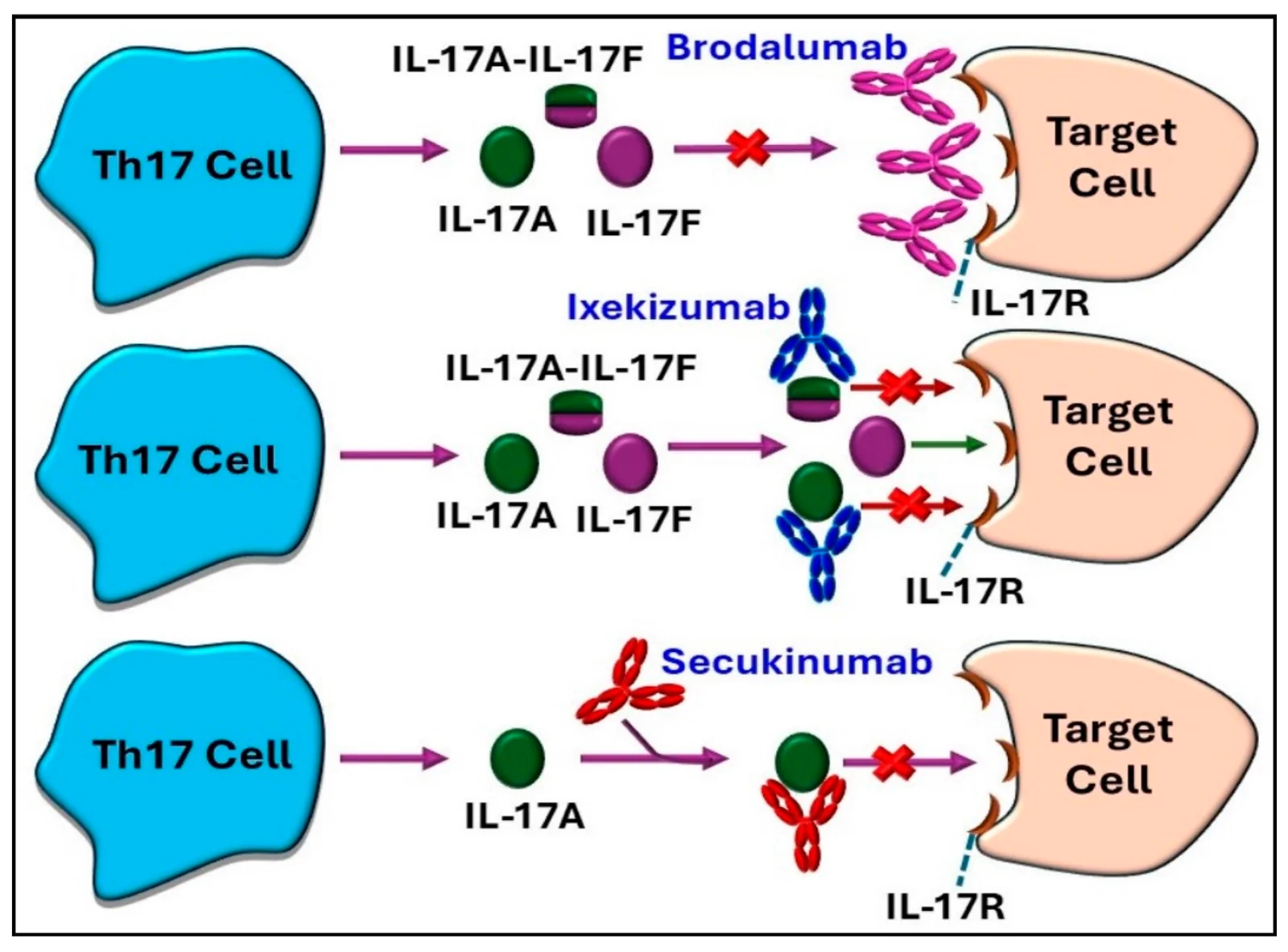
Mechanisms of action of biologics targeting interleukin-17 (IL-17) and the IL-17 receptor (IL-17R) among proinflammatory cytokines and mediators.

The IL-17 pathway plays a central role in the pathogenesis of several autoimmune and inflammatory diseases by promoting the recruitment of neutrophils and the production of pro-inflammatory cytokines, chemokines, and antimicrobial peptides [227]. Targeting this pathway has therefore emerged as a strategic therapeutic approach for conditions such as psoriasis, psoriatic arthritis, ankylosing spondylitis, and non-radiographic axial spondyloarthritis. Biologic agents directed against IL-17 or its receptor, including secukinumab and ixekizumab (IL-17A inhibitors) and brodalumab (IL-17 receptor A antagonist), have demonstrated robust efficacy in clinical trials, resulting in rapid reduction in inflammation, disease activity, and associated clinical symptoms [228,229,230]. These therapies represent a significant advancement in immunomodulatory treatment by specifically inhibiting a key pro-inflammatory axis while sparing broader immune functions (Figure 5) (Table 1 and Table 2).

##### Brodalumab (BRO)

Brodalumab is a fully human IgG2 monoclonal antibody originally developed by Amgen and subsequently licensed to Valeant Pharmaceuticals (now Bausch Health) for further development and commercialization. It specifically targets the IL-17 receptor A (IL-17RA), thereby blocking signaling from several IL-17 family cytokines, including IL-17A, IL-17F, and IL-17E. By inhibiting receptor-mediated signaling, brodalumab suppresses downstream pro-inflammatory pathways implicated in autoimmune and inflammatory diseases [231,232]. Brodalumab received U.S. FDA approval in February 2017 under the brand name Siliq^®^ for the treatment of moderate-to-severe plaque psoriasis in adult patients who are candidates for systemic therapy or phototherapy. By preventing IL-17 cytokines from engaging their receptor, brodalumab effectively reduces keratinocyte hyperproliferation and cutaneous inflammation associated with psoriatic lesions [233].

##### Ixekizumab (IXE)

Ixekizumab is a humanized IgG4 monoclonal antibody that selectively binds to IL-17A, neutralizing its activity and preventing receptor engagement on target cells. By inhibiting IL-17A signaling, ixekizumab reduces inflammatory responses mediated by Th17 cells and related immune pathways. It is indicated for the treatment of moderate-to-severe plaque psoriasis and is under evaluation in additional inflammatory conditions in ongoing clinical trials. Phase II clinical trials have demonstrated its capacity to significantly attenuate IL-17-driven inflammation, supporting its clinical efficacy in psoriasis management [234,235,236].

##### Secukinumab (SEC)

Secukinumab is a fully human recombinant IgG1κ monoclonal antibody that selectively neutralizes IL-17A. It binds both the IL-17A homodimer (IL-17A/A) and the IL-17A/F heterodimer, preventing their interaction with the IL-17 receptor complex (IL-17RA/RC) on target cells. IL-17A is produced predominantly by Th17 CD4+ T cells, as well as by CD8+ T cells (Tc17), neutrophils, and mast cells, playing a central role in driving tissue inflammation and autoimmunity [230,237,238]. Clinical evaluation of secukinumab in a phase II trial demonstrated robust efficacy in plaque psoriasis. Administration of a single 3 mg/kg dose resulted in a 63% reduction in the Psoriasis Area and Severity Index (PASI), highlighting its potential to significantly mitigate disease activity by targeting IL-17A-mediated pathways [239].

#### 5.4.4. Anti-IL-12, IL-23, IL-23p19, and IL-22 Biologics

**Figure 6:**
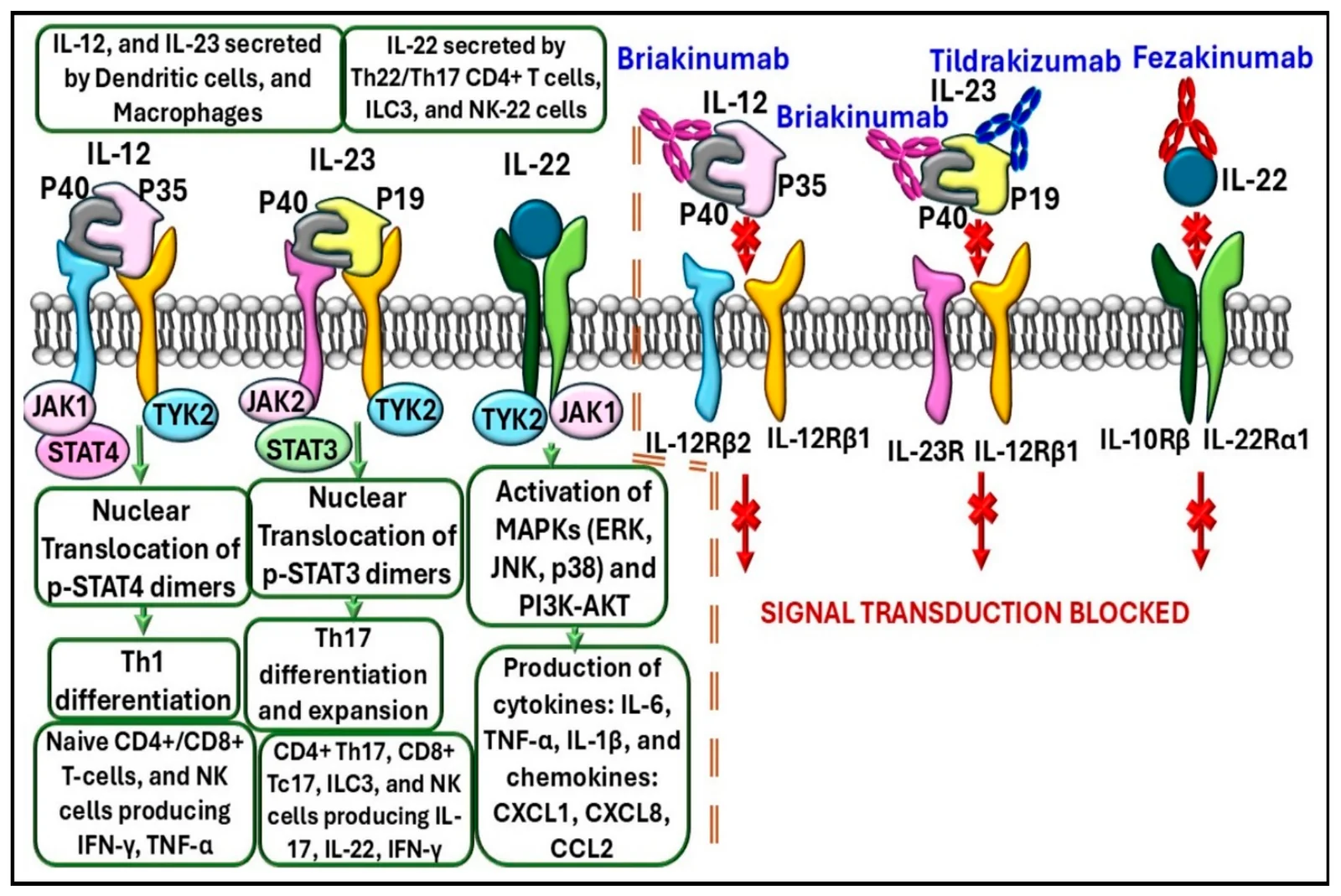
Mechanisms of action of biologics targeting interleukin-12 (IL-12), IL-23, IL-23p19 subunit, and IL-22 within the proinflammatory cytokine and mediator pathways.

The IL-12 and IL-23 cytokines are pivotal regulators of immune responses, particularly in Th1- and Th17-mediated inflammation [240]. IL-12 is a heterodimeric cytokine composed of p35 and p40 subunits, whereas IL-23 shares the p40 subunit with IL-12 but contains a distinct p19 subunit. These cytokines drive differentiation and activation of Th1 and Th17 cells, respectively, resulting in the production of downstream inflammatory mediators such as interferon-γ (IFN-γ), IL-17, and IL-22, which contribute to the pathogenesis of autoimmune and chronic inflammatory diseases, including psoriasis, psoriatic arthritis, and inflammatory bowel disease. Therapeutic targeting of these cytokines through monoclonal antibodies that selectively neutralize IL-12, IL-22, IL-23, or their specific subunits [241,242,243]. By selectively interfering with critical cytokine signaling axes, these biologics provide a focused anti-inflammatory strategy, attenuating pathogenic immune responses while minimizing broad immunosuppression in autoimmune and chronic inflammatory conditions (Figure 6) (Table 1 and Table 2).

##### Briakinumab (BRI)

Briakinumab is a fully human recombinant IgG1 monoclonal antibody that targets the p40 subunit shared by IL-12 and IL-23. By binding this subunit, briakinumab neutralizes both cytokines (IL-12 and IL-23), preventing their interaction with the IL-12Rβ1 receptor on T cells. This blockade inhibits downstream signaling pathways that drive Th1- and Th17-mediated immune responses, ultimately reducing the inflammatory processes central to psoriasis pathogenesis [244].

In a double-blind, placebo-controlled phase II trial, 180 patients with moderate-to-severe plaque psoriasis were randomized to receive various regimens of ABT-874 (briakinumab) or placebo. At 12 weeks, PASI 75 responses were achieved by 63% (200 mg single dose), 93% (100 mg every other week), 90% (200 mg every other week), 90% (200 mg weekly), and 3% in the placebo group. At 24 weeks, maintenance of PASI 75 was observed in 23% (200 mg single dose), 60% (100 mg every other week), 60% (200 mg weekly for four doses), 73% (200 mg every other week), 83% (200 mg weekly), and 7% of the placebo group [116,245,246]. Despite these promising results, development of briakinumab was discontinued in 2011 after phase III trials revealed increased risks of serious infections and cardiovascular events, including myocardial infarction and stroke.

##### Fezakinumab (FEZ)

Fezakinumab (ILV-094) is a fully human monoclonal antibody that selectively targets IL-22, a cytokine involved in epidermal hyperproliferation and skin barrier dysfunction. Pfizer Inc. (New York, NY, USA) initiated clinical trials for ILV-094 in conditions including psoriasis, atopic dermatitis, and rheumatoid arthritis [247]. In phase II studies, fezakinumab combined with methotrexate demonstrated significant improvements in the Scoring Atopic Dermatitis (SCORAD) index in patients with severe atopic dermatitis, highlighting its potential to modulate IL-22-driven inflammation. However, as of the latest available information, fezakinumab has not received regulatory approval and is not commercially marketed [248].

##### Tildrakizumab (TIL)

Tildrakizumab is a fully humanized IgG1κ monoclonal antibody that selectively binds to the p19 subunit of IL-23, preventing its interaction with the IL-23 receptor on T cells and dendritic cells. IL-23 is a key driver of Th17 cell differentiation and proliferation, leading to the secretion of pro-inflammatory cytokines such as IL-17, which contribute to psoriasis and other autoimmune conditions [249].

Tildrakizumab is indicated for the treatment of moderate-to-severe plaque psoriasis in adults, particularly in patients who are candidates for systemic therapy or phototherapy and in those who have not responded or are intolerant to other systemic treatments. Subcutaneous administration of tildrakizumab has been shown to reduce disease severity and improve skin lesions in clinical studies. The drug was originally developed by Schering-Plough Corporation (Kenilworth, NJ, USA) and later acquired by Merck & Co. (Kenilworth, NJ, USA). Later, Sun Pharmaceuticals (Mumbai, India) obtained global marketing rights in 2014 and markets it as Ilumya^®^ [250].

##### Ustekinumab (UST)

Ustekinumab is a fully human monoclonal antibody that targets the p40 subunit shared by IL-12 and IL-23, thereby blocking the activity of these pro-inflammatory cytokines. By inhibiting both the Th1 and Th17 immune pathways, ustekinumab provides an effective therapeutic option for patients with moderate-to-severe plaque psoriasis. The drug was approved by the U.S. FDA and is marketed under the trade name Stelara^®^ (Janssen Biotech, Inc., a subsidiary of Johnson & Johnson, headquartered in Horsham, PA, USA) [251].

The PHOENIX 2 phase III trial, a multicenter, randomized, placebo-controlled, double-blind study, evaluated 1230 patients receiving subcutaneous doses of ustekinumab 45 mg, 90 mg, or placebo at weeks 0 and 4, followed by dosing every 12 weeks for a total of 28 weeks. At week 12, PASI 75 was achieved by 67% (45 mg), 76% (90 mg), and 4% (placebo) [252]. Placebo patients who crossed over to ustekinumab treatment at week 12 showed substantial improvement, with sustained PASI 90 responses observed through week 28 [116]. The efficacy of ustekinumab underscores the critical role of IL-23/Th17 signaling in psoriasis pathogenesis, wherein undifferentiated T-helper cells exposed to TGF-β and IL-6 differentiate into Th17 cells, subsequently producing IL-6 and IL-17 [253].

#### 5.4.5. Anti-IL-6 and IL-6 Receptor Targeted Biologics

Interleukin-6 (IL-6) is a pleiotropic cytokine with a central role in inflammation and immune regulation. It is produced by a variety of cell types, including macrophages, fibroblasts, endothelial cells, and T cells. IL-6 mediates acute-phase responses, promotes B-cell differentiation, induces T-cell proliferation, and facilitates the differentiation of naïve CD4+ T cells into Th17 cells in the presence of transforming growth factor-β (TGF-β). Dysregulated IL-6 signaling contributes to chronic inflammation and is implicated in numerous autoimmune and inflammatory diseases, such as rheumatoid arthritis, systemic lupus erythematosus, and giant cell arteritis [254].

IL-6 exerts its biological effects via classical signaling and trans-signaling. Classical signaling occurs when IL-6 binds to the membrane-bound IL-6 receptor (mIL-6R) and associates with the signal-transducing subunit gp130, activating downstream pathways including JAK/STAT3, MAPK, and PI3K/Akt. Trans-signaling occurs when soluble IL-6 receptor (sIL-6R) forms a complex with IL-6, which activates gp130 on cells that do not express mIL-6R, thereby broadening the range of IL-6-responsive cells [255].

Therapeutic inhibition of IL-6 signaling has been achieved through monoclonal antibodies targeting either IL-6 itself or the IL-6 receptor (IL-6R). Anti-IL-6 antibodies—namely Clazakizumab, Elsilimomab, MED15117, Olokizumab, Sirukumab, and Ziltivekimab—bind circulating IL-6 and inhibit its interaction with both mIL-6R and sIL-6R. In contrast, anti-IL-6R antibodies—namely Tocilizumab, Sarilumab, and Vobarilizumab—block IL-6 from engaging its receptor, thereby inhibiting both classical and trans-signaling pathways [256] (Table 1 and Table 2).

##### Clazakizumab (CLA)

Clazakizumab (BMS-945429; ALD518) is a humanized monoclonal antibody directed against IL-6, a pivotal cytokine in immune regulation and inflammation. Initially developed by Alder Biopharmaceuticals (King of Prussia, PA, USA) and later acquired by CSL Behring in 2020, clazakizumab remains an investigational therapy without regulatory approval. Its mechanism of action involves binding to IL-6 and blocking its interaction with both soluble and membrane-bound IL-6 receptors, thereby inhibiting downstream signaling cascades that mediate inflammatory responses and tissue damage. This makes it a potential therapeutic option for IL-6-driven conditions, including rheumatoid arthritis (RA), psoriatic arthritis, and antibody-mediated kidney transplant rejection [257].

In a randomized, double-blind, phase IIb clinical trial, clazakizumab achieved its primary endpoint by significantly reducing disease activity in patients with moderate-to-severe RA who had an inadequate response to TNF inhibitors. Additional Phase II trials in psoriatic arthritis confirmed musculoskeletal benefits, although without a consistent dose–response relationship. Furthermore, clazakizumab demonstrated promise in chronic active antibody-mediated rejection following kidney transplantation. Despite these encouraging findings, the agent has not advanced to regulatory approval and continues to be under clinical investigation [258,259].

##### Elsilimomab (ELS)

Elsilimomab (B-E8) is a murine IgG1 monoclonal antibody developed by Diaclone (France) that neutralizes soluble IL-6. By binding to IL-6, it prevents receptor engagement and downstream activation of signaling pathways, including JAK/STAT, MEK/ERK, and PI3K/Akt. This blockade disrupts IL-6-mediated mechanisms implicated in the pathogenesis of inflammatory disorders and hematological malignancies. Despite evidence of biological activity in preclinical and early clinical studies, Elsilimomab did not progress to commercialization and has no approved trade name [260].

##### Olokizumab (OLO)

Olokizumab (CP6038; Artlegia^®^) is a humanized IgG4κ monoclonal antibody that selectively neutralizes IL-6 by binding to site 3 of the cytokine, thereby preventing its interaction with the gp130 co-receptor. This blockade disrupts both classical (membrane-bound IL-6R-mediated) and trans-signaling (soluble IL-6R-mediated) pathways, resulting in attenuation of IL-6-driven inflammation and downstream tissue injury associated with diseases like RA [261].

Olokizumab has been evaluated extensively in the CREDO clinical trial program, which confirmed its efficacy and safety in patients with RA, particularly in combination with methotrexate in individuals with inadequate responses to prior therapies. In May 2020, olokizumab received regulatory approval in Russia for the treatment of RA and was later approved in Kazakhstan, Belarus, Kyrgyzstan, and Azerbaijan. It was also approved for COVID-19 management in Russia. However, it has not been approved by either the U.S. FDA or the EMA [262].

##### Sarilumab (SAR)

Sarilumab is a fully human monoclonal antibody directed against the interleukin-6 receptor (IL-6R), binding both the soluble (sIL-6R) and membrane-bound (mIL-6R) isoforms. By blocking the IL-6/IL-6R interaction, sarilumab prevents recruitment of the gp130 signaling subunit and inhibits downstream pro-inflammatory pathways, including JAK/STAT, MAPK/ERK, and PI3K/Akt cascades, which are central to rheumatoid arthritis (RA) pathogenesis. Clinically, sarilumab is approved for the treatment of moderate-to-severe RA in adults with an inadequate response or intolerance to conventional disease-modifying antirheumatic drugs (DMARDs). Its therapeutic effects include suppression of C-reactive protein (CRP), inhibition of T-cell activation, and modulation of B-cell differentiation, thereby reducing systemic inflammation and joint damage [263].

Sarilumab (Kevzara^®^) was co-developed by Sanofi (Paris, France) and Regeneron Pharmaceuticals Inc. (NY, USA), with its first regulatory approval granted by the U.S. Food and Drug Administration (FDA) in 2017 for adult patients with moderate-to-severe RA [264]. The pivotal TARGET trial, a randomized, double-blind, placebo-controlled Phase III study, evaluated sarilumab in RA patients with inadequate response or intolerance to anti-TNF therapies. The results demonstrated significant improvements in clinical and laboratory measures of disease activity, confirming sarilumab’s efficacy in patients refractory to biologic therapies [265].

##### Sirukumab (SRK)

Sirukumab is a fully human monoclonal antibody (IgG1κ) that specifically targets and binds to IL-6, a cytokine involved in the pathogenesis of RA. By binding to IL-6, sirukumab prevents its interaction with soluble and membrane-bound IL-6 receptors on target cells, thereby inhibiting the downstream signaling pathways that contribute to inflammation. It is used in the treatment of moderate-to-severe RA [266,267].

Sirukumab underwent several Phase III clinical trials for RA, including the SIRROUND-D, -H, and -T studies, which demonstrated its efficacy in reducing signs and symptoms of RA. Despite these advancements, in 2017, the FDA Advisory Committee did not recommend approval of sirukumab for the treatment of moderate to severe RA. In long-term safety and efficacy studies, SRK demonstrated safety concerns, including an increased risk of major adverse cardiovascular events [268]. Following the FDA’s decision, Johnson and GlaxoSmithKline (GSK) decided to discontinue the development of sirukumab for RA [269].

##### Tocilizumab (TCZ)

Tocilizumab is a recombinant, humanized IgG1 monoclonal antibody with immunomodulatory properties that selectively targets the IL-6R. It binds with high affinity to both sIL-6R and mIL-6R receptor isoforms, thereby competitively inhibiting IL-6 from associating with its receptor. This inhibition prevents the subsequent recruitment and dimerization of the signal-transducing glycoprotein 130 (gp130) subunit, a critical step required for activation of downstream signaling cascades, including the JAK/STAT, MAPK/ERK, and PI3K/Akt pathways [267,270]. IL-6 is a pleiotropic cytokine that orchestrates immune regulation, hematopoiesis, and acute-phase responses. While crucial for host defense, IL-6 overproduction drives pathogenic inflammation and systemic hyperactivation syndromes, including cytokine release syndrome and autoimmune disorders such as RA, sJIA, polyarticular JIA, giant cell arteritis (GCA), and systemic sclerosis-associated interstitial lung disease [271].

Tocilizumab was developed to neutralize IL-6-mediated pathology by simultaneously targeting sIL-6R and mIL-6R. Structurally, it is a humanized IgG1 monoclonal antibody with the canonical H2L2 polypeptide configuration, consisting of two identical heavy chains (H2) and two identical light chains (L2). Its therapeutic mechanism is based on competitive inhibition of IL-6 signaling, thereby attenuating inflammation, regulating bone remodeling, and reducing immune-mediated tissue damage [272].

Clinical trials have demonstrated that TCZ improves clinical signs, reduces disease activity, and prevents radiographic progression in RA [273,274]. Long-term data from the Safety and Tolerability of Tocilizumab Monotherapy for Rheumatoid Arthritis (STREAM) study demonstrated sustained efficacy and safety of TCZ over a five-year period, with superior clinical benefits observed at the 8 mg/kg dose. TCZ significantly reduced acute-phase markers (CRP, ESR), improved hemoglobin levels through suppression of the iron-regulatory hormone hepcidin, and stabilized systemic inflammatory activity [275]. However, immunogenicity-related adverse events, including anaphylaxis and anaphylactoid reactions, were reported more frequently at the lower doses of TCZ (4 mg/kg) when administered without concomitant methotrexate, likely due to the development of anti-TCZ antibodies [276].

Beyond systemic effects, TCZ modulates the behavior of synovial fibroblast-like synoviocytes (FLS), key mediators of RA pathology. It upregulates long non-coding RNA MIR31HG expression while downregulating microRNA-214 (miR-214), thereby restoring PTEN activity and inhibiting AKT-driven proliferation, migration, and matrix metalloproteinase (MMP) release [277]. Additionally, TCZ promotes macrophage polarization toward an anti-inflammatory phenotype and protects chondrocytes from catabolic and inflammatory damage. TCZ demonstrated efficacy in glucocorticoid-dependent polymyalgia rheumatica [278] and in RA patients with life-threatening complications such as hyperferritinemic syndrome and capillary leak syndrome (CLS), particularly in cases refractory to glucocorticoids and high-dose intravenous immunoglobulin (IVIG) therapy [279]. It also reduces complement components C3 and C4, highlighting its role in systemic inflammation [280]. Generally, glucocorticoids are considered the gold standard for preventing severe vascular complications; however, their use is associated with substantial morbidity and mortality. In a Phase II study of patients with new-onset or relapsing giant cell arteritis (GCA), intravenous TCZ (8 mg/kg) administered every 4 weeks for 52 weeks effectively induced and maintained disease remission. By week 12, patients achieved normalization of erythrocyte sedimentation rate (ESR) and C-reactive protein (CRP) and were largely asymptomatic on a tapering prednisolone dose of 0.1 mg/kg/day [281,282,283].

IL-6 contributes to neuronal hyperexcitability and epileptogenesis through the activation of pro-inflammatory signaling pathways. TCZ has shown therapeutic efficacy in refractory status epilepticus (RSE) and febrile infection-related epilepsy syndrome (FIRES), where it reduces NF-kB activity and downstream inflammatory mediators such as prostaglandin E2 (PGE2) and cyclooxygenase-2 (COX-2) [192,284,285]. In a prospective, randomized controlled study in patients with RSE, adjunctive treatment with TCZ significantly lowered serum NF-κB levels, which are implicated in inflammation-induced neurodegeneration, as well as other pro-inflammatory cytokines [284,286]. Elevated IL-6 expression in both cerebrospinal fluid (CSF) and plasma has been strongly correlated with convulsive epilepsy, where it plays a pivotal role in generating neuronal hyperexcitability and modulating absence seizure activity. Experimental data further support this mechanism: IL-6 blockade with TCZ reduced the incidence of absence seizures and ameliorated depressive-like comorbidities in WAG/Rij rats [287].

Furthermore, AA amyloidosis is a serious complication of chronic inflammatory disorders, arising from the deposition of amyloid fibrils derived from serum amyloid A protein (SAA). Therapeutic strategies, therefore, focus on reducing SAA levels to prevent further amyloid deposition. TCZ therapy in patients with treatment-refractory chronic inflammatory conditions, including cases complicated by AA amyloidosis and in renal transplant recipients, led to a rapid decline in SAA concentrations (from 70 mg/L to 4 mg/L) within 10 days of the first dose, with suppression maintained for up to 23 months [288]. In patients with RA, TCZ modulates angiogenesis by significantly reducing circulating extracellular matrix metalloproteinase inducer (EMMPRIN/CD147), while simultaneously increasing the expression of circulating microRNAs miR-146a-5p and miR-150-5p. This is accompanied by a concomitant decrease in pro-angiogenic mediators, including vascular endothelial growth factor (VEGF) and matrix metalloproteinase-9 (MMP-9) [289].

The cytokine storm can activate the coagulation cascade, induce vascular endothelial cell dysfunction, and impair myocardial contractility [290]. In patients with rheumatoid arthritis, tocilizumab (TCZ) therapy improved arterial stiffness, as evidenced by reduced pulse wave velocity (PWV) and lowered brachial blood pressure in an atherosclerosis model [186]. Moreover, TCZ has demonstrated potential to attenuate both the initiation and progression of atherosclerosis, while also promoting stabilization of atherosclerotic plaques [291,292]. Preclinical and clinical evidence further suggests that TCZ may mitigate ischemia–reperfusion injury and limit adverse left ventricular remodeling following myocardial infarction [293,294]. In a clinical trial of patients with non-ST-elevation myocardial infarction (NSTEMI), administration of a single intravenous dose of TCZ prior to coronary angiography significantly reduced circulating levels of high-sensitivity C-reactive protein (hsCRP) and high-sensitivity troponin T (hsTnT) during the first three days of hospitalization [295]. These findings indirectly suggest that IL-6 blockade with TCZ may attenuate myocardial inflammation and confer cardioprotective effects in patients with NSTEMI [296].

Tocilizumab (TCZ) has demonstrated efficacy in refractory dermatological and autoimmune conditions, including psoriasis, psoriatic arthritis, Behçet’s disease, systemic lupus erythematosus, systemic sclerosis, relapsing polychondritis, vasculitis, and atopic dermatitis [297]. It is also effective in non-infectious uveitis, a potentially sight-threatening inflammation of the uveal tract [298]. In experimental periodontitis, systemic TCZ administration (2–8 mg/kg) attenuated alveolar bone resorption and attachment loss, reduced inflammatory cell infiltration, and suppressed Th17-related cytokines and RANKL expression [299]. Additionally, TCZ promotes an anti-inflammatory monocyte/macrophage phenotype via upregulation of PPARγ [300]. Additionally, in RA patients, TCZ therapy has been associated with reduced systemic inflammation and attenuation of chronic kidney disease (CKD) progression [301]. During the COVID-19 pandemic, TCZ gained emergency approval for hospitalized adults and children with severe disease, due to its ability to mitigate cytokine release syndrome [302,303]. Combination therapy with corticosteroids further improved survival in critically ill patients. Severe combined immunodeficiency (SCID) patients with BCG vaccination undergoing allogeneic hematopoietic stem cell transplantation (HSCT) developed BCG-related inflammatory syndromes (BCG-IS), which were controlled by IL-1 and IL-6 inhibitors [304].

##### Vobarilizumab (VOB)

Vobarilizumab is a humanized bispecific nanobody that selectively targets both soluble (sIL-6R) and membrane-bound (mIL-6R) forms of the IL-6R. By binding to IL-6R, vobarilizumab competitively inhibits IL-6 from interacting with its receptor, thereby suppressing downstream pro-inflammatory signaling cascades, including JAK/STAT, MAPK/ERK, and PI3K/Akt pathways [305]. Consequently, vobarilizumab attenuates systemic and tissue-specific inflammation, reducing immune-mediated tissue damage in autoimmune diseases such as RA and SLE. Phase IIb clinical trials demonstrated that vobarilizumab exhibited efficacy comparable to that of tocilizumab (Actemra) in RA patients. Despite these promising results regarding its therapeutic potential, efficacy, and safety profile in autoimmune disorders, vobarilizumab has not received regulatory approval and is not commercially available [306].

##### Ziltivekimab (ZIL)

Ziltivekimab is a fully human IgG1 monoclonal antibody directed against IL-6, a pleiotropic cytokine implicated in the pathogenesis of autoimmune, inflammatory, and cardiovascular diseases. By binding directly to soluble IL-6, Ziltivekimab prevents its interaction with both membrane-bound and soluble IL-6 receptor, thereby suppressing downstream pro-inflammatory signaling cascades, including the JAK/STAT, MAPK/ERK, and PI3K/Akt pathways. Through this mechanism, Ziltivekimab disrupts IL-6-mediated processes implicated in systemic inflammation, immune dysregulation, and hepcidin-driven anemia of chronic disease [307].

During its developmental history, Ziltivekimab has been designated under several code names. It was initially developed as MEDI5117 by MedImmune/AstraZeneca (Gaithersburg, MD, USA), later referred to as COR-001 in subsequent clinical development programs, and subsequently as ABBV-181 following acquisition by AbbVie Inc. (North Chicago, IL, USA) and continued development. Ultimately, it was assigned the International Nonproprietary name (INN) Ziltivekimab [308].

As MEDI5117 (AstraZeneca/MedImmune), Ziltivekimab underwent a first-in-human, randomized, double-blind, placebo-controlled Phase I clinical trial in patients with rheumatoid arthritis. This study evaluated safety, tolerability, pharmacokinetics, pharmacodynamics, and immunogenicity and demonstrated an acceptable safety profile with evidence of biological activity. Despite these favorable findings, AstraZeneca discontinued development prior to Phase II/III trials, and MEDI5117 did not progress to regulatory submission or approval [309].

Under AbbVie’s sponsorship (ABBV-181/Ziltivekimab), the biologic advanced to Phase II clinical trials for autoimmune and inflammatory disorders, including RA, SLE, psoriasis, Crohn’s disease, and ulcerative colitis. More recently, the developmental emphasis has expanded into cardiovascular and renal indications, particularly anemia of inflammation and chronic kidney disease (CKD), in which IL-6-mediated hepcidin dysregulation contributes to morbidity [310]. In the RESCUE Phase II trial (NCT03926117), Ziltivekimab significantly reduced systemic inflammatory biomarkers such as high-sensitivity C-reactive protein, fibrinogen, and serum amyloid A in patients with CKD and elevated cardiovascular risk [311]. Building on these results, the ongoing ZEUS Phase III cardiovascular outcomes trial (NCT05021835) is evaluating Ziltivekimab for the reduction in major adverse cardiovascular events in patients with CKD and systemic inflammation. As of 2025, Ziltivekimab has not received regulatory approval. Its clinical significance lies in its mechanistic differentiation from IL-6 receptor antagonists such as tocilizumab and sarilumab [312].

### 5.5. Biologics Targeting B-Cell Pathways (B-Cell Depletion or Survival Inhibitors)

B cells are critical mediators of adaptive immunity, contributing to immune homeostasis through antibody production, antigen presentation, and cytokine secretion. Dysregulation of B-cell function, including autoantibody production and abnormal survival, plays a central role in the pathogenesis of various autoimmune and chronic inflammatory diseases such as SLE, RA, and multiple sclerosis (MS). Consequently, targeting B-cell pathways has emerged as a key therapeutic strategy to mitigate pathogenic immune responses while preserving essential immune functions [313].

**Figure 7:**
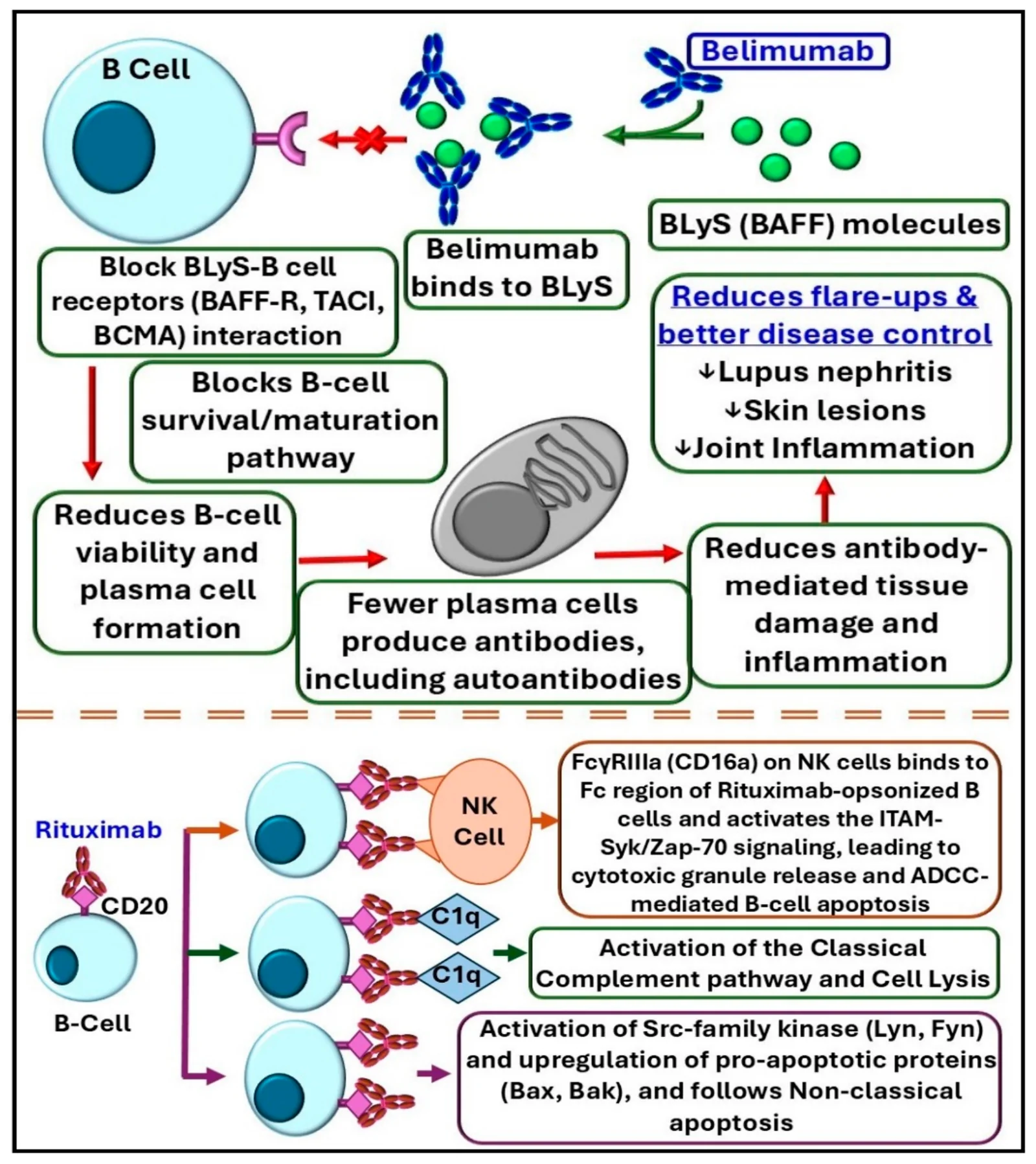
Mechanisms of action of biologics targeting B-cell activation, signaling, and survival pathways.

Biologics targeting B cells operate primarily through two mechanisms: depletion of pathogenic B cells and inhibition of B-cell survival. By modulating B-cell activity at multiple levels, these biologics offer precision-based intervention capable of reducing disease activity, preventing organ damage, and improving long-term outcomes in patients with autoimmune and inflammatory diseases [314] (Figure 7) (Table 1 and Table 2).

#### 5.5.1. Belimumab (BEL)

Belimumab is a fully human IG1λ monoclonal antibody that selectively binds to soluble B-lymphocyte stimulator (BLyS), also known as B-cell activating factor (BAFF), a cytokine of the TNF superfamily critical for B-cell survival, differentiation, and activation [315,316]. By neutralizing BLyS, belimumab inhibits its interaction with B-cell receptors, thereby reducing the survival of autoreactive B cells and limiting pathogenic autoantibody production. Elevated BLyS levels have been implicated in autoimmune disorders, including systemic lupus erythematosus (SLE), by promoting the persistence of autoreactive B-cell populations [317].

Belimumab functions as a B-cell survival inhibitor, rather than a direct depleting agent. It blocks soluble BLyS, preventing downstream signaling that would otherwise enhance the proliferation and differentiation of autoreactive B cells. This immunomodulatory mechanism reduces systemic inflammation and tissue damage in autoimmune disease without complete B-cell depletion [318]. Belimumab is primarily indicated for autoimmune disorders, including SLE and lupus nephritis (LN). It is used as an add-on therapy in adults with active disease despite standard-of-care treatment, particularly in patients with positive anti-dsDNA antibodies and evidence of disease activity [319,320]. Clinical effects typically manifest within 6–12 weeks, with efficacy closely linked to baseline BLyS levels. In 2020, belimumab became the first FDA-approved biologic for non-renal SLE, demonstrating low immunogenicity and a favorable safety profile in clinical trials [321,322].

The BLISS-52 and BLISS-76 Phase III trials—multinational, randomized, double-blind studies—demonstrated that belimumab reduced SLE disease activity, flare rates, and corticosteroid requirements, while extending time to first flare compared to placebo [323,324]. The drug was generally well-tolerated, with common adverse events including headache, upper respiratory tract infection, arthralgia, nausea, urinary tract infection, diarrhea, fatigue, and pyrexia occurring at rates below 10% [325].

Post hoc analyses from these trials indicated that belimumab ameliorated renal involvement, reducing renal flares and improving renal activity parameters compared to placebo [326]. More recently, the BLISS-LN trial confirmed the efficacy and safety of belimumab in active LN, showing that patients receiving belimumab alongside standard therapy achieved complete renal response (CRR) at week 104, with improvements in proteinuria, renal function, and overall disease activity [327].

Novel BLyS and BLyS/APRIL (a proliferation-inducing ligand) pathway inhibitors, such as telitacicept, have demonstrated efficacy in patients with refractory autoimmune manifestations, including lupus hepatitis unresponsive to conventional therapy and prior belimumab treatment [328,329]. Other agents targeting the BLyS pathway, such as atacicept, are also under clinical investigation, underscoring the therapeutic potential of B-cell survival inhibition in autoimmune diseases.

#### 5.5.2. Rituximab (RTX)

Rituximab (IDEC-C288), a chimeric human-mouse IgG1 monoclonal antibody that selectively targets CD20, a surface marker expressed on mature B cells, leading to B-cell depletion via complement-dependent cytotoxicity (CDC) and antibody-dependent cellular cytotoxicity (ADCC). By eliminating CD20-positive B cells, rituximab reduces the production of pathogenic autoantibodies and modulates immune-mediated inflammatory processes [315,330]. Rituximab exerts a direct B-cell depleting effect on abnormal B-cell populations while indirectly diminishing autoantibody-mediated tissue damage. This dual action mitigates systemic inflammation in autoimmune disorders and immune-mediated inflammatory diseases characterized by dysregulated humoral responses, including RA, SLE, granulomatosis with polyangiitis (GPA), and microscopic polyangiitis (MPA) [315]. In RA, rituximab has demonstrated sustained clinical improvement in patients with active disease despite methotrexate (MTX) therapy. Open-label studies, including those by Edwards et al. (2004) [331], confirmed that selective B-cell depletion leads to significant reductions in disease activity. Rituximab is approved in the United States for the treatment of moderate-to-severe RA in combination with MTX, for patients with an inadequate response to one or more anti-TNF-α inhibitors [331].

In SLE, rituximab has shown efficacy in patients refractory to conventional therapies, including those with refractory lupus nephritis, autoimmune cytopenias, or neuropsychiatric manifestations [332,333]. However, rituximab is not formally approved for SLE and is generally employed as an off-label option in severe, treatment-resistant cases. Rituximab is typically administered intravenously using two standard regimens: (i) 375 mg/m^2^ once weekly for four consecutive weeks in combination with corticosteroids and/or other immunosuppressive agents, or (ii) 1 g on days 1 and 15, repeated every six months, as employed in the EXPLORER Phase II/III trial [334,335]. Rituximab therapy can significantly impair humoral responses, as demonstrated by reduced antibody titers to the pandemic H1N1 influenza vaccine [336]. Other monoclonal antibodies targeting CD20, such as ocrelizumab and ofatumumab, exert comparable B-cell-depleting effects and are either approved or under clinical investigation for various autoimmune and hematologic disorders.

### 5.6. Biologics Targeting IgE and Allergy-Modulating Pathways

Immunoglobulin E (IgE) is traditionally recognized for its central role in mediating allergic responses through activation of mast cells, basophils, and other effector immune cells. However, emerging evidence indicates that IgE also contributes to the pathogenesis of certain chronic inflammatory and autoimmune diseases by promoting aberrant immune activation, cytokine release, and tissue inflammation. Elevated IgE levels have been reported in conditions such as SLE, atopic dermatitis with autoimmune features, and chronic spontaneous urticaria with systemic inflammation, suggesting that IgE-mediated pathways may extend beyond classical allergic diseases [337,338,339,340,341].

Biologics that target IgE or modulate IgE-driven immune responses offer a precision-based therapeutic approach to attenuate inflammation and autoimmunity. Biologics such as omalizumab, ligelizumab, and quilizumab represent a shift toward immune pathway-specific interventions, targeting upstream mediators of inflammation rather than solely addressing symptomatic manifestations [342,343] (Table 1 and Table 2).

#### 5.6.1. Omalizumab (OMA)

Omalizumab (E25/OMZ) is a recombinant humanized IgG1κ monoclonal antibody that specifically targets IgE. By binding to the Cε3 domain of free IgE, omalizumab prevents its interaction with the high-affinity IgE receptor (FcεRI) on mast cells and basophils, thereby reducing receptor expression and attenuating downstream allergic inflammatory responses [344]. It is manufactured by Genentech, Inc. (CA, USA) in collaboration with Novartis Pharmaceuticals Corporation (NJ, USA) under the brand name Xolair^®^. It is used primarily in the management of allergic asthma and other allergic conditions by binding to IgE and preventing its activation of allergic responses. This mechanism not only sequesters circulating IgE but also diminishes effector cell activation, leading to decreased release of pro-inflammatory mediators [345].

Commercially developed by Genentech Inc. (CA, USA) in collaboration with Novartis Pharmaceuticals (NJ, USA), omalizumab is marketed under the trade name Xolair^®^. Clinically, it is approved for the treatment of moderate-to-severe persistent allergic asthma, chronic spontaneous urticaria, and other IgE-mediated disorders [346]. By neutralizing IgE and suppressing effector cell activation, omalizumab provides a targeted therapeutic approach for patients with refractory allergic disease. Novel IgE-targeting agents, including ligelizumab and quilizumab, are under investigation to enhance efficacy, prolong therapeutic effects, and broaden applications in autoimmune and systemic inflammatory disorders.

MEDI-4212 (developmental code) is a high-affinity, humanized anti-IgE monoclonal antibody developed by MedImmune/AstraZeneca. It binds soluble IgE at epitopes spanning the Cε3/Cε4 domains, thereby preventing interaction with both the high-affinity receptor FcεRI (expressed on mast cells, basophils, and dendritic cells) and the low-affinity receptor CD23 [347]. This mechanism results in direct neutralization of circulating free-IgE, achieving reductions approximately 1.0 log greater than those observed with omalizumab. Importantly, MEDI-4212 effectively lowered free IgE concentrations near 1 IU/mL, a level predicted to provide optimal suppression of IgE-mediated responses in patients with severe asthma [348,349]. By rapidly depleting free IgE, MEDI-4212 downregulates FcεRI expression on effector cells, thereby attenuating IgE-mediated cellular activation. In addition, engineered MEDI-4212 variants with enhanced Fc effector functions demonstrated ADCC against IgE-expressing memory B cells in preclinical studies [350]. In a phase I, randomized, placebo- and omalizumab-controlled, first-in-human, dose-escalation study in atopic subjects, MEDI-4212 showed greater potency than omalizumab, with more rapid and profound suppression of serum-free IgE. Treatment also led to reduced FcεRI expression on basophils and dendritic cells, and was generally well tolerated, with adverse events of mostly low severity [347].

#### 5.6.2. Ligelizumab

Ligelizumab is a humanized IgG1κ monoclonal antibody that binds to the Cε3 domain of IgE with substantially higher affinity than omalizumab. By cross-linking across the IgE-Fc dimer, ligelizumab achieves more complete neutralization of circulating IgE and more potent inhibition of IgE binding to the high-affinity receptor FcεRI on effector cells such as mast cells and basophils. This results in greater suppression of basophil activation in both preclinical and clinical studies [351].

In early clinical investigations, Arm et al. (2014) [352] demonstrated that ligelizumab, compared with omalizumab, induced higher suppression of free serum IgE, including in patients with high baseline IgE levels. Pharmacodynamic effects included stronger inhibition of allergen-induced skin prick test responses. These findings were consistent with preclinical assessments and randomized, double-blind, placebo-controlled trials in atopic individuals [352].

The pivotal PEARL 1 and PEARL 2 Phase III trials were multicenter, randomized, double-blind, placebo- and active-controlled studies to evaluate the efficacy and safety of ligelizumab in adolescent and adult patients with chronic spontaneous urticaria (CSU) inadequately controlled by H1-antihistamines. The primary objective was to demonstrate superior efficacy compared to omalizumab (Xolair). Results showed that ligelizumab provided meaningful clinical benefit, with efficacy and safety outcomes in adolescents comparable to those observed in adults [353,354,355,356].

Based on these results, ligelizumab received Breakthrough Therapy designation from the U.S. FDA in January 2021 for the treatment of CSU in patients with inadequate response to H1-antihistamines [357]. However, as of 2025, ligelizumab has not received FDA marketing authorization for any indication. Collectively, these findings establish ligelizumab as a next-generation anti-IgE therapy with superior capacity for neutralizing free IgE and more potent inhibition of FcεRI-mediated effector cell activation compared with omalizumab.

#### 5.6.3. Quilizumab (Anti M1 Prime Monoclonal Antibody)

Quilizumab (MEMP-1972A, RG-7449, sometimes reported as h47H4) is an afucosylated, humanized IgG1 monoclonal antibody that selectively targets the M1-prime epitope of membrane-bound IgE (mIgE) expressed on IgE-committed B cells. This M1-prime epitope is present on the B-cell receptor of IgE-switched memory B cells but absent on circulating soluble IgE, thereby enabling quilizumab to deplete IgE-expressing B cells without directly neutralizing soluble IgE [358]. Elevated serum levels of total and allergen-specific IgE are strongly associated with atopic diseases such as allergic rhinitis and allergic asthma. While conventional anti-IgE antibodies (e.g., omalizumab) act by neutralizing free IgE, antibodies directed against the M1-prime epitope suppress IgE production by eliminating IgE-committed B cells. Quilizumab was evaluated in Phase I and II trials assessing safety, pharmacokinetics, and pharmacodynamics in subjects with allergic rhinitis (NCT01160861) and mild allergic asthma (NCT01196039) [359].

Afucosylation of the Fc domain enhances quilizumab’s affinity for FcγRIIIa on effector cells, thereby increasing ADCC against mIgE-positive B cells. This mechanism results in depletion of IgE-expressing B cells and reduced generation of new IgE [358]. Pharmacodynamic data demonstrated sustained reductions in both total and allergen-specific IgE (approximately 30–40%) for several months following administration. This mechanism is distinct from omalizumab, which neutralizes soluble IgE, and ligelizumab, which binds soluble IgE with higher affinity but does not directly deplete IgE-committed B cells [359].

Despite its robust biomarker effects, quilizumab failed to demonstrate clinically meaningful efficacy. In a large, randomized phase II study in patients with allergic asthma, reductions in serum IgE did not translate into significant improvements in exacerbation rates, lung function, or symptom control [360]. Similar outcomes were observed in phase I studies in allergic rhinitis [361]. As a result, the clinical development of quilizumab was discontinued in 2015, and it has not received regulatory approval from either the U.S. FDA or EMA [362].

## 6. Pharmacovigilance and Systemic Tolerability of Immunobiologics

While the therapeutic efficacy of immunobiologics is well established, their systemic immunomodulatory mechanisms inherently introduce safety considerations, primarily concerning an increased susceptibility to infections. Tumor necrosis factor (TNF) inhibitors, as a broad class, are associated with an elevated risk of both serious and opportunistic infections, including the reactivation of latent tuberculosis and herpes zoster. However, meaningful pharmacological differences exist within this class. A comprehensive 2024 network meta-analysis encompassing 61 randomized controlled trials (RCTs) and 20,458 patients demonstrated that certolizumab pegol significantly increased the risk of serious infection compared with placebo, and that certolizumab pegol and infliximab carried a higher risk of serious infection relative to other TNF inhibitors within the network. By contrast, etanercept and golimumab were associated with the lowest comparative infection risk within the class [363]. Epidemiological data indicate that this infection risk peaks during the first six months of therapy and is substantially amplified by confounding clinical variables, including concomitant glucocorticoid administration, advanced age, diabetes mellitus, and smoking. Consequently, universal screening for latent tuberculosis and hepatitis B prior to the initiation of any biologic therapy remains a mandatory clinical protocol to mitigate these risks.

Beyond infectious complications, the potential for biologic therapy to alter immune surveillance and influence oncogenesis remains a primary imperative of pharmacovigilance. Overall, the risk of solid-tumor malignancies does not appear broadly elevated across all biologic classes; rather, the risk profile is highly specific to the mechanistic class and the individual agent. For instance, prolonged exposure to TNF inhibitors is consistently associated with a modestly increased risk of non-melanoma skin cancer. Furthermore, a large 2024 cohort study consisting of 25,305 individuals revealed that B-cell depleting therapies, specifically rituximab, were associated with a higher incident cancer risk relative to TNF inhibitors (hazard ratio [HR], 1.91), followed by the T-cell costimulation modulator abatacept (HR, 1.47) [364]. To contextualize these clinical findings, the ORAL Surveillance trial demonstrated that Janus kinase (JAK) inhibitors carry a comparatively higher risk for both incident malignancies and major adverse cardiovascular events than TNF inhibitors, substantiating the rationale for preserving biologics as the preferred first-line advanced therapy in distinct patient populations [365]. Interestingly, cytokine-specific blockade can yield divergent oncological signals, namely exploratory analyses from the CANTOS cardiovascular outcomes trial found that interleukin-1β (IL-1β) inhibition via canakinumab was associated with a reduction in incident and fatal lung cancer, a hypothesis-generating signal that prompted the subsequent CANOPY program of trials in non-small-cell lung cancer; however, the phase III CANOPY-A adjuvant trial did not meet its primary checkpoint, underscoring that this potential anti-tumor association remains unconfirmed in a dedicated oncology population [366,367].

A fundamental pharmacokinetic challenge associated with protein-based therapeutics is immunogenicity, characterized by the generation of anti-drug antibodies (ADAs). ADAs emerge through both T-cell-dependent and T-cell-independent recognition of non-human or neo-epitope sequences within the biologic structure. ADA development inversely correlates with the degree of molecular humanization; murine (-omab) and chimeric (-ximab) monoclonal antibodies inherently carry a higher immunogenic risk compared to humanized (-zumab) or fully human (-umab) constructs. The clinical consequences of robust ADA generation are significant, often leading to accelerated drug clearance, secondary loss of therapeutic response, and, less frequently, severe infusion or hypersensitivity reactions, including anaphylaxis, which has been documented in a small subset of patients with detectable anti-tocilizumab antibodies [368,369].

Distinct from classical ADA-mediated immunogenicity, some biologics carry idiosyncratic, off-target immune-mediated toxicities; daclizumab (Zinbryta), for example, was withdrawn from the market following reports of immune-mediated encephalitis and serious hepatotoxicity unrelated to anti-drug antibody formation [370,371]. To circumvent immunogenicity-related loss of response, clinical mitigation strategies frequently employ the co-administration of conventional disease-modifying antirheumatic drugs (csDMARDs) like methotrexate [372]. At the molecular level, immunogenicity is actively addressed through advanced Fc engineering, complete sequence humanization, and PEGylation, exemplified by certolizumab pegol, which lacks an Fc region entirely. Furthermore, proactive therapeutic drug monitoring and emerging nanotechnology-based tolerization strategies are increasingly utilized to sustain long-term drug efficacy [373].

In addition to class-wide evaluations, certain biologics exhibit distinct, idiosyncratic adverse event profiles that determine their therapeutic positioning in clinical practice [374]. Etanercept, for example, has been implicated in autoantibody induction resulting in drug-induced lupus erythematosus [375,376]. More severely, therapies that indiscriminately block leukocyte trafficking into the central nervous system, notably natalizumab and the discontinued efalizumab, are associated with progressive multifocal leukoencephalopathy (PML) driven by JC virus (John Cunningham virus) reactivation [377,378]. These safety profiles directly inform contemporary guideline-based clinical decision-making. Guidelines established by the ACR, EULAR, and AGA/ECCO conventionally position TNF inhibitors as the preferred first-line biologic following the failure of conventional immunosuppressants, while reserving IL-17 and IL-23 inhibitors for patients exhibiting inadequate responses or specific contraindications [379,380,381,382,383]. Precision medicine principles increasingly guide these selections; for instance, gut-selective vedolizumab is heavily favored over systemically acting natalizumab in inflammatory bowel disease to circumvent PML risk entirely [384]. Similarly, abatacept may be preferentially selected for patients presenting with elevated cardiovascular risk due to its favorable cardiovascular safety profile, while seropositive rheumatoid arthritis phenotypes strongly favor rituximab intervention [385]. Ultimately, the selection of an immunobiologic is a complex, shared decision-making process that must synthesize baseline biomarker status, administration logistics, biosimilar availability, and comprehensive risk stratification.

## 7. The Biosimilar Paradigm: Regulatory Frameworks, Interchangeability, Pharmacoeconomic Impact and Future

The patent expiration of clinical-grade reference immunobiologics has facilitated the development of biosimilars, establishing a vital pathway for healthcare cost mitigation and broader therapeutic availability. In the United States, this regulatory framework was established under the Biologics Price Competition and Innovation Act (BPCIA) of 2010, which instituted an expedited approval pathway for biosimilars [386]. Under these guidelines, the investigational biologic should exhibit high structural and functional similarity to the reference originator product, demonstrating no clinically meaningful differences in safety profile, purity, or biological potency. Rather than evaluating biosimilars through traditional de novo phase III efficacy trials, both the FDA and EMA base regulatory approval on a “totality-of-evidence” approach accumulated across analytical, non-clinical, and clinical dimensions. This paradigm prioritizes extensive structural and functional characterization, pharmacokinetic and pharmacodynamic (PK/PD) bioequivalence, and comparative clinical immunogenicity assessments to confirm therapeutic equivalence. While the EMA utilizes an equivalent centralized procedure, international regulatory approaches regarding the authorization and utilization of biosimilars have recently undergone significant convergence. Initially, the widespread adoption of biosimilars was hindered by varying regulatory standards regarding interchangeability, a regulatory status that permits automatic substitution at the pharmacy without the explicit authorization of the prescribing clinician.

For years, the FDA required manufacturers to conduct dedicated, highly controlled switching studies to obtain an interchangeable designation, a regulatory barrier the EMA and the Heads of Medicines Agencies (HMA) addressed in 2022 through a joint statement declaring that all EU-authorized biosimilars are scientifically interchangeable with their reference products, without the need for additional systematic switching studies [387,388,389].

In June 2024, the FDA issued draft guidance proposing to eliminate the switching-study requirement for interchangeability designation. This shift was informed by an FDA-led systematic review and meta-analysis of 31 switching studies (encompassing 44 distinct switch treatment periods across 21 biosimilars), which found no clinically meaningful differences in the risk of death, serious adverse events, or treatment discontinuation between switched and non-switched patient cohorts, findings that reflect a broader global regulatory consensus with the EMA. Since that guidance, interchangeable biosimilar approvals have accelerated markedly, with the majority of all U.S. interchangeable biosimilars approved since 2024, including several adalimumab biosimilars [390,391].

The integration of biosimilars into clinical practice is primarily driven by pharmacoeconomics. Market entry of biosimilars acts as a critical regulatory and economic driver across the United States and European healthcare systems, successfully lowering the benchmark prices of reference biologics such as adalimumab, infliximab, and etanercept. Consequently, the expansion of the interchangeable biosimilar ecosystem is projected to substantially reduce both direct acquisition costs and broader administration-related healthcare expenditures. This economic paradigm shift is particularly impactful for low- and middle-income countries (LMICs), where the high capital requirements of originator biologics have systematically limited patient access to advanced targeted therapies, ultimately expanding equity and availability of disease-modifying treatments (DMTs) for severe autoimmune and inflammatory conditions [392].

Despite these regulatory and economic advancements, systemic barriers to widespread clinical and market adoption persist. Continued impediments to widespread utilization include clinician and patient unfamiliarity with the FDA regulatory mechanisms for biosimilar approval, alongside state-level pharmacy substitution statutes that remain unaligned with federal interchangeability designations. Furthermore, manufacturer rebate structures and formulary exclusivity contracts consistently mitigate price competition following biosimilar market entry. Clinical adoption is also constrained by residual practitioner apprehension regarding non-medical switching in immune-mediated diseases, despite the robust postmarketing immunogenicity and safety data documented [393,394]. The future of the biosimilar market requires cooperation between multiple stakeholders beyond price competition. Moreover, regulators and developers should collaborate to ensure biosimilars reach patients rapidly without compromising stringent quality, safety, or efficacy standards.

## 8. Conclusions and Future Perspectives

Therapeutic immunobiologics have revolutionized the treatment of autoimmune and autoinflammatory diseases/disorders by enabling targeted modulation of key pathogenic immune pathways. Compared with broad-spectrum immunosuppressants, these agents have demonstrated superior efficacy and, in many cases, improved safety profiles. Therapeutic agents such as T-cell costimulation blockers (e.g., abatacept), B-cell depleting or -modulating agents (e.g., rituximab, belimumab), pro-inflammatory cytokines inhibitors (anti-TNF, anti-IL-6, anti-IL-17/23 families), or leukocyte trafficking inhibitors (integrin antagonists such as vedolizumab and natalizumab) exemplify the spectrum of mechanisms that have delivered substantial clinical benefit across diverse autoimmune and inflammatory conditions, including rheumatoid arthritis, inflammatory bowel disease (IBD), psoriasis, systemic lupus erythematosus and beyond.

Despite these advances, critical challenges remain. Disease heterogeneity, variability in therapeutic response kinetics, treatment-associated immunogenicity, increased risk of infections, and substantial economic burden continue to restrict the widespread application of biologic therapies. The differential efficacy observed between biologic-naïve and biologic-experienced patients, together with variability across distinct disease endotypes and organ systems, underscores the need for more precise strategies in patient selection and the development of personalized treatment paradigms.

Future progress will depend on the development of predictive biomarkers that can inform therapeutic response, durability, and safety. Integrative multi-omic approaches, including genomics, transcriptomics, proteomics, and metabolomics, combined with detailed clinical phenotyping, hold promise for biomarker-guided treatment optimization and evidence-based de-escalation strategies. For patients who fail to respond to monotherapy, rationally designed combination regimens, incorporating dual biologics that target complementary immune pathways with or without adjunctive small molecules, require rigorous evaluation to establish additive efficacy, with vigilant monitoring for potential safety risks.

By enabling highly targeted modulation of immune pathways, immunobiologics have transformed the management of autoimmune and inflammatory diseases, resulting in improved efficacy and safety profiles compared with conventional therapies. Despite their clinical success, challenges related to delivery, immunogenicity, long-term safety, and interindividual variability remain critical considerations. Emerging strategies, including alternative administration routes, advanced formulation and delivery technologies, precision targeting, and biomarker-guided patient stratification, hold promise for further enhancing therapeutic outcomes. Inhaled biologics represent a promising non-invasive alternative to parenteral administration with potential for both localized and systemic immunomodulation. Advances in aerosol engineering, particle design, and formulation technologies can help to overcome barriers related to stability, deposition, and pulmonary absorption of therapeutic proteins and peptides. A comprehensive understanding of lung physiology and biopharmaceutical interactions will be essential to optimize pharmacokinetics, minimize variability, and ensure long-term safety. Future translational and clinical investigation should focus on scalable delivery platforms and long-term outcome evaluation to establish inhaled immunobiologics as viable modalities in next-generation biologics therapy [395].

Advances in molecular engineering also represent a critical frontier of innovation. Strategies aimed at reducing immunogenicity, including Fc modifications, PEGylation, and optimized dosing paradigms, have the potential to improve therapeutic durability. Furthermore, next-generation modalities, including bispecific antibodies, antibody-drug conjugates, receptor fusion proteins, and agents engineered for enhanced tissue specificity, are poised to broaden the therapeutic armamentarium. Long-term pharmacovigilance remains essential to detect delayed or rare adverse events and to inform real-world safety assessments. In parallel, the development and global availability of high-quality biosimilars are critical for enhancing cost-effectiveness and broadening equitable access to these transformative therapies. In conclusion, therapeutic immunobiologics constitute a cornerstone of contemporary immunotherapy for autoimmune and inflammatory diseases. Continued advancements in precision medicine, biomarker-guided therapy, molecular and antibody engineering, and health-system integration are expected to drive substantial progress over the next decade, further optimizing therapeutic efficacy, safety, accessibility, and equity in clinical practice.

## Figures and Tables

**Table 1:** Biologics for autoimmune and inflammatory diseases: Names, Development code, Type/class, mechanism of action, brand names, FDA/EMA approval, and manufacturer details.

	Biologic	Developmental Code	Type/Class	Mechanism of Action	Brand Name	FDA/EMA Approval	Manufacturer and Address
1	Abatacept (ABA)	CTLA4-Ig	Fusion protein (CTLA-4-IgG1)	Binds CD80/CD86, blocks T-cell co-stimulation	Orencia^®^	2005/2007	Bristol-Myers Squibb Company, New York, NY, USA
2	Basiliximab (BAS)	SAB-1	Chimeric murine/human mAb (IgG1κ)	Targets IL-2Rα (CD25)	Simulect^®^	1998/1998	Novartis Pharmaceuticals Corporation, East Hanover, NJ, USA
3	Daclizumab (DAC)	D2E7	Humanized mAb (IgG1)	Targets IL-2Rα (CD25)	Zenapax^®^	2016/2016	Genentech (a member of the Roche Group), South San Francisco, CA, USA (Discontinued in 2018)
4	Alefacept (ALE)	LFA3-Ig	Fusion protein (LFA-3-IgG1 Fc)	CD2/LFA-3 inhibitor (Inhibitor of T-cell activation)	Amevive^®^	2003/-	Astellas Pharma US, Inc., Deerfield, IL, USA (discontinued it in 2011 due to declining demand)
5	Siplizumab (SIP)	MEDI-507	Humanized mAb (human IgG1κ)	CD2/LFA-3 inhibitor	No Trade Name	Not approved	MedImmune (AstraZeneca), Gaithersburg, MD, USA (discontinued during clinical trials in early 2003 without marketing).
6	Efalizumab (EFA)	hu-1124	Humanized mAb (IgG1κ)	Targets CD11a (LFA-1)	Raptiva^®^	2003/2004	Genentech, Inc., South San Francisco, CA, USA (discontinued in 2009 due to the risk of progressive multifocal leukoencephalopathy (PML)
7	Etrolizumab (ETR)	rhuMAb Beta 7; RG-7413	Humanized mAb (IgG1κ)	Targets β7 subunit of integrins (blocks both α4β7 and αEβ7)	No Trade Name	Not approved	Genentech (a member of the Roche group)
8	Natalizumab (NAG)	AN100226	Humanized mAb (IgG4κ)	Targets α4 integrins. Blocks interaction with VCAM-1 and MAdCAM-1	Tysabri^®^	2004/2006 (Multiple sclerosis); 2008/2007 (Crohn’s disease)	Biogen Inc., Cambridge, MA, USA
9	Vedolizumab (VDZ)	MLN0002	Humanized mAb (IgG1)	Targets α4β7 integrin	Entyvio^®^	2014/2014	Takeda Pharmaceuticals, Cambridge, MA, USA
10	Adalimumab (ADA)	D2E7	Fully human mAb (IgG1)	Anti-TNFα (TNF-alpha inhibitor)	Humira^®^	2002/2003	AbbVie Inc., North Chicago, IL, USA
11	Certolizumab pegol (CZP)	CDP870	PEGylated humanized Fab’ fragment	Anti-TNFα, lacks Fc region	Cimzia^®^	2008/2009	UCB Pharma, Atlanta, GA, USA
12	Etanercept (ETN)	TNFR:Fc	Fusion protein (TNFR2-Fc)	Anti-TNFα (TNF-alpha inhibitor)	Enbrel^®^	1998/2000	Amgen Inc., Thousand Oaks, CA, USA. Pfizer Inc., New York, NY, USA.
13	Golimumab (GOL)	CNTO 148	Fully human mAb (IgG1κ)	Anti-TNFα (TNF-alpha inhibitor)	Simponi^®^	2009/2009	Janssen Biotech, Inc., Horsham, PA, USA
14	Infliximab (IFX)	cA2	Chimeric mAb (IgG1κ)	Anti-TNFα (TNF-alpha inhibitor)	Remicade^®^	1998/1999	Schering-Plough (acquired by Merck & Co., Kenilworth, NJ, USA) and Centocor (a subsidiary of Johnson & Johnson) Janssen Biotech, Inc., Horsham, PA, USA
15	Anakinra (ANR)	IL-1ra	Recombinant IL-1 receptor antagonist (rhIL-1Ra)	Anti-IL-1 agent (IL-1 receptor antagonist (IL-1Ra))	Kineret^®^	2001/2002	Swedish Orphan Biovitrum, Stockholm, Sweden
16	Canakinumab (CAN)	ACZ885	Fully human mAb (IgG1κ)	Anti-IL-1β (inhibits IL-1β)	Ilaris^®^	2009/2009	Novartis Pharmaceuticals Corporation, East Hanover, NJ, USA
17	Rilonacept (RLC)	NLR-09	Dimeric Fusion protein (IL-1R/IL-1RAcP-Fc)	IL-1 Trap	Arcalyst^®^	2008/2009	Regeneron Pharmaceuticals, Inc., Tarrytown, NY, USA
18	Brodalumab (BRO)	AMG 827	Fully human mAb (IgG2)	Anti-IL-17Ra (Targets IL-17Ra)	Siliq^®^(U.S.) Kyntheum (E.U.), Lumicef (Japan)	2017/2017	Amgen (Thousand Oaks, CA, USA); AstraZeneca (Cambridge, UK); Kyowa Hakko Kirin (Tokyo, Japan) Valeant Pharmaceuticals (now Bausch Health, Bridgewater, NJ, USA).
19	Ixekizumab (IXE)	LY2439821	Humanized mAb (IgG4)	Anti-IL-17A (Targets IL-17A)	Taltz (Torutsu, Japan)	2016/2016	Eli Lilly & Co., Indianapolis, IN, USA
20	Secukinumab (SEC)	AIN-457	Fully human mAb (IgG1κ)	Anti-IL-17A (Targets IL-17A)	Cosentyx^®^	2015/2015	Novartis Pharmaceuticals Corporation, East Hanover, NJ, USA
21	Briakinumab (BRI)	ABT-874	Human mAb (IgG1κ)	Anti-P40 subunit of IL-12 and IL-23 (Targets IL-12/23 (p40 subunit)	No Trade name	Not approved	Abbott, Abbott Park, IL (now AbbVie Inc., North Chicago, IL, USA) (discontinued in 2011 after phase 3 trials)
22	Fezakinumab (FEZ)	ILV-094	Human mAb (IgG1)	Anti-IL-22 (Targets Il-22)	No Trade Name	Not approved	Pfizer Inc., New York, NY, USA (discontinued in its development phase in 2017)
23	Tildrakizumab (TIL)	SCH900222	Humanized mAb (IgG1κ)	Anti-IL-23 (Targets p19 subunit of IL-23)	Ilumya^®^ (U.S.) Ilumetri (E.U.)	2018/2018	Sun Pharmaceuticals Industries, Ltd., Kandivli (East), Mumbai, India
24	Ustekinumab (UST)	CNTO-1275	Human mAb (IgG1κ)	Anti-P40 (Targets p40 subunit of IL-12/23)	Stelara^®^	2009/2009	Janssen Biotech, Inc., Horsham, PA, USA
25	Clazakizumab (CLA)	BMS-945429 & ALD518	Humanized mAb (IgG1κ)	Targets IL-6	No Trade Name	Not approved	Developed by Alder Biopharmaceuticals, King of Prussia, PA, USA, later acquired by CSL Behring in 2020.
26	Elisilimomab (ELS)	B-E8	Murine mAb (IgG1κ)	Anti-IL-6	No Trade Name	Not approved	Diaclone, Besancon, France
27	Olokizumab (OLO)	CDP6038	Humanized mAb (IgG4κ)	Targets IL-6 (site 3, blocks gp130 interaction)	Artlegia	Not approved	R-Pharm Group (Moscow, Russia)
28	Sarilumab (SAR)	REGN88 or SAR153191	Fully human mAb (IgG1)	Targets IL-6R	KevZara^®^	2017/2017	Regeneron Pharmaceuticals Inc. (Tarrytown, NY, USA) & Sanofi (Paris, France)
29	Sirukumab (SRK)	CNTO-136	Human mAb (IgG1κ)	Targets IL-6	Plivensia^®^	Not approved	Developed by Centocor (now Janssen Biotech, Inc.), Harrisburg, PA, USA, a subsidiary of Johnson & Johnson
30	Tocilizumab (TCZ)	R-1569	Humanized mAb (IgG1κ)	Targets IL-6R	Actemra^®^ (U.S.) RoActemra (E.U.)	2010/2009	Chugai Pharmaceutical Co., Ltd. (a member of Roche Group), Otsu-shi, Shiga, Japan
31	Vobarilizumab (VOB)	ALX-0061	Humanized bispecific nanobody (IL-6R targeting)	Targets IL-6R	No trade name	Not approved	Ablynx NV (Ghent, Belgium), a subsidiary of Sanofi S.A. (Paris, France)
32	Ziltivekimab (ZIL)	MEDI5117, COR-001, ABBV-181	Human mAb (IgG1)	Targets IL-6 (site 1)	In development phase	Not approved	AbbVie Inc., North Chicago, IL, USA (formerly known as Abbott Laboratories)
33	Belimumab (BEL)	LymphoStat-B	Fully human mAb (IgG1λ)	Targets BLyS (BAFF)	Benlysta^®^	2011/2011	GlaxoSmithKline (GSK), Middlesex, UK
34	Rituximab (RTX)	IDEC-C28B	Chimeric human-mouse mAb (IgG1κ)	Targets CD20	Rituxan^®^ (U.S.) MabThera^®^ (E.U.)	1997/1998	Genentech, Inc., South San Francisco, CA, USA, and Roche Group, Basel, Switzerland
35	Omalizumab (OMA)	E25/OMZ	Humanized mAb (IgG1κ)	Targets IgE	Xolair^®^	2003/2005	Genentech, Inc., South San Francisco, CA, USA, and Novartis Pharmaceuticals Corporation, East Hanover, NJ, USA
36	Ligelizumab (LIG)	QGE031	Humanized mAb (IgG1κ)	Targets IgE	No Trade name	Not approved	Novartis/Celgene/Genentech
37	Quilizumab (QUI)	MEMP-1972A, RG-7449	Afucosylated humanized mAb (IgG1)	Targets the M1-prime epitope of membrane-bound IgE	No trade name	Not approved	Genentech/Roche

**Table 2:** Biologics with therapeutic applications for various autoimmune and inflammatory diseases.

S. No.	Drugs	Therapeutics for Autoimmune and Inflammatory Diseases
1	Abatacept	Rheumatoid arthritis, juvenile idiopathic arthritis, psoriatic arthritis, Crohn’s disease, psoriasis, and ulcerative colitis
2	Adalimumab	Rheumatoid arthritis, Crohn’s disease, psoriasis, and ulcerative colitis
3	Alefacept	Psoriasis
4	Anakinra	Autoinflammatory diseases including Cryopyrin-associated periodic syndromes (CAPS) and COVID-19-related complications
5	Basiliximab	Organ transplantation
6	Belimumab	Systemic lupus erythematosus (SLE) and lupus nephritis
7	Briakinumab	Autoimmune diseases such as psoriasis
8	Brodalumab	Moderate-to-severe plaque psoriasis
9	Canakinumab	Autoinflammatory diseases including CAPS, systemic juvenile idiopathic arthritis (SJIA) and other inflammatory conditions and cardiovascular diseases
10	Certolizumab Pegol	Rheumatoid arthritis, Crohn’s disease, ankylosing spondylitis, psoriatic arthritis, and non-radiographic axial spondyloarthritis
11	Clazakizumab	Rheumatoid arthritis, psoriatic arthritis, lupus nephritis, kidney transplant rejection
12	Daclizumab	Organ transplantation and autoimmune diseases
13	Efalizumab	Moderate-to-severe psoriasis
14	Elsilimomab	Rheumatoid arthritis, multiple myeloma, cancers
15	Etanercept	Rheumatoid arthritis, psoriatic arthritis, ankylosing spondylitis, and plaque psoriasis
16	Etrolizumab	Ulcerative colitis, Crohn’s disease (IBD)
17	Fezakinumab	Psoriasis and other inflammatory conditions
18	Golimumab	Rheumatoid arthritis, psoriatic arthritis, ankylosing spondylitis, and ulcerative colitis
19	Infliximab	Rheumatoid arthritis, Crohn’s disease, ankylosing spondylitis, psoriatic arthritis, and ulcerative colitis
20	Ixekizumab	Psoriasis, psoriatic arthritis, axial spondyloarthritis
21	Ligelizumab	Chronic spontaneous urticaria (CSU), asthma, food allergy
22	Natalizumab	Multiple sclerosis, Crohn’s disease
23	Olokizumab	Rheumatoid arthritis
24	Omalizumab	Moderate-to-severe persistent asthma, chronic idiopathic urticaria (chronic hives), and certain allergic conditions
25	Quilizumab	Allergic asthma, allergic rhinitis
26	Rilonacept	CAPS and other inflammatory conditions, including some rare diseases like Familial Mediterranean Fever (FMF)
27	Rituximab	Rheumatoid arthritis and certain cancers such as non-Hodgkin lymphoma and chronic lymphocytic leukemia (CLL)
28	Sarilumab	Rheumatoid arthritis, polymyalgia rheumatica
29	Secukinumab	Psoriasis, ankylosing spondylitis, psoriatic arthritis, and non-radiographic axial spondyloarthritis
30	Siplizumab	Graft-versus-host disease (GVHD) and psoriasis
31	Sirukumab	Rheumatoid arthritis
32	Tildrakizumab	Psoriasis and psoriatic arthritis
33	Tocilizumab	Rheumatoid arthritis, cytokine release syndrome, systemic juvenile idiopathic arthritis, and giant cell arteritis
34	Ustekinumab	Psoriasis, psoriatic arthritis, and Crohn’s disease
35	Vedolizumab	Ulcerative colitis, Crohn’s disease
36	Vobarilizumab	Rheumatoid arthritis, SLE
37	Ziltivekimab	Rheumatoid arthritis, SLE, psoriasis, Crohn’s disease, and ulcerative colitis

## Data Availability

No new data were created or analyzed in this study.
